# Impaired hepatic autophagy exacerbates hepatotoxin induced liver injury

**DOI:** 10.1038/s41420-023-01368-3

**Published:** 2023-02-21

**Authors:** Katherine Byrnes, Niani Tiaye Bailey, Kamal Baral, Arissa Mercer, Spandan Joshi, Nickol Wahby, Tyler Rorison, Gang Liu, Xiao-Ming Yin, Bilon Khambu

**Affiliations:** Department of Pathology and Laboratory Medicine, Tulane University School of Medicine, New Orleans, LA 70112 USA

**Keywords:** Pathogenesis, Hepatotoxicity

## Abstract

Hepatotoxins activate the hepatic survival pathway, but it is unclear whether impaired survival pathways contribute to liver injury caused by hepatotoxins. We investigated the role of hepatic autophagy, a cellular survival pathway, in cholestatic liver injury driven by a hepatotoxin. Here we demonstrate that hepatotoxin contained DDC diet impaired autophagic flux, resulting in the accumulation of p62-Ub-intrahyaline bodies (IHBs) but not the Mallory Denk-Bodies (MDBs). An impaired autophagic flux was associated with a deregulated hepatic protein-chaperonin system and significant decline in Rab family proteins. Additionally, p62-Ub-IHB accumulation activated the NRF2 pathway rather than the proteostasis-related ER stress signaling pathway and suppressed the FXR nuclear receptor. Moreover, we demonstrate that heterozygous deletion of Atg7, a key autophagy gene, aggravated the IHB accumulation and cholestatic liver injury. *Conclusion*: Impaired autophagy exacerbates hepatotoxin-induced cholestatic liver injury. The promotion of autophagy may represent a new therapeutic approach for hepatotoxin-induced liver damage.

## Introduction

Liver is the primary organ that metabolizes and excretes liver toxins. Hepatotoxins or their reactive metabolites and intermediaries injure cells and tissue, causing liver dysfunction. When exposed to hepatotoxins, host cells activate survival mechanisms such as macroautophagy, hereafter referred to as autophagy, to minimize their damaging effects. Although extensive research has been conducted on hepatotoxin-mediated cell death (apoptosis and necrosis), little attention has been paid to how host survival mechanisms might mitigate hepatotoxin-induced tissue damage and death.

Autophagy is an intracellular lysosomal degradative pathway widely known for nutrient turnover, organelle, or signaling protein quality control. During autophagy, proteins and organelles are wrapped in double-membrane vesicles called autophagosomes [1, 2]. The autophagosomes then fuse with the lysosome to form autolysosomes and then use lysosomal degradative enzymes to degrade the cellular contents sequestered in the autophagosomes. The formation of misfolded protein aggregates is a common response to hepatotoxins exposure [3]. By clearing these aggregates, autophagy aids in cellular quality control and ameliorates cellular damage.

DDC (3,5-diethoxycarbonyl-1,4-dihydrocollidine) is the most widely used animal model to study hepatotoxin-induced cholestatic liver injury, ductular reaction, and fibrosis [4]. Chronic feeding of DDC has long been used to study the formation of Mallory-Denk bodies (MDBs) [5], primary sclerosing cholangitis (PSC) [6], porphyria [6, 7], chronic cholangiopathies [8], biliary fibrosis [6, 8], and ductular reactions [6, 9].

Using an acute DDC diet model of hepatotoxins-induced cholestatic liver injury, we demonstrate that autophagy flux is impaired, and this leads to an accumulation of hepatic proteins such as p62 and Ub, which leads to intrahyaline bodies (IHB) formation. The accumulation of p62-Ub-containing IHB in the liver activates the NRF2 pathway and downregulates the FXR nuclear receptor, resulting in the deregulation of bile acid (BA). Additionally, cholestatic liver injury and IHB formation are exacerbated by impaired autophagy. Thus, autophagy protects against DDC diet toxicity by regulating the formation of p62-Ub IHBs and modulating p62-NRF2-FXR linked cholestatic liver injury.

## Results

### Acute DDC intoxication leads to accumulation of p62 and Ub containing intracytoplasmic hyaline bodies (IHB) but not Mallory-Denk bodies (MDBs) in liver

In the liver, millions of protein molecules are synthesized and secreted each day, including plasma proteins [10, 11]. Proteotoxic effects may occur when abnormalities occur in this process, either through the gain or loss of function of intracellular proteins. Acute feeding of hepatotoxin such as DDC, cause hepatic proteinopathy [5, 6]. Hepatic proteinopathy is followed by liver injury and other histological features of common liver diseases. However, how the DDC diet causes abnormal hepatic protein accumulation, and its pathogenic consequences remain unclear.

**Fig. 1:**
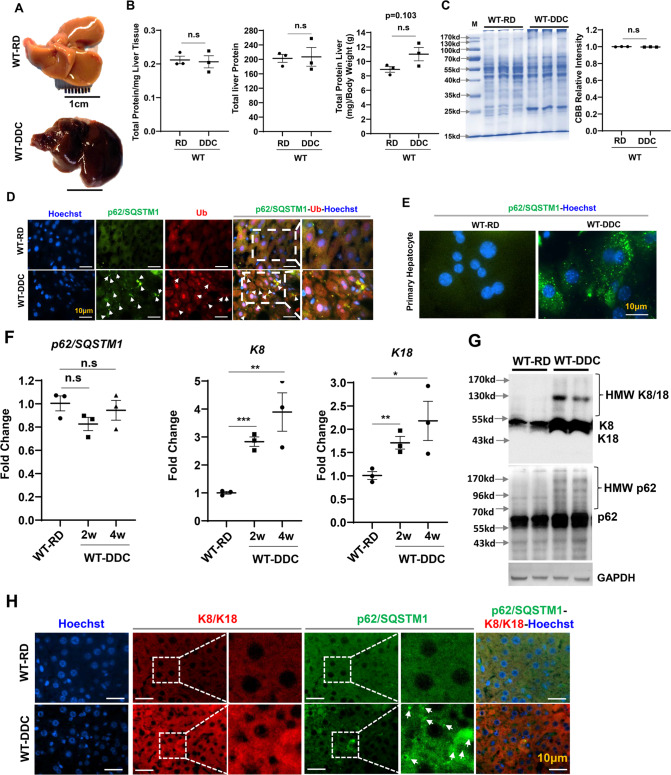
Hepatic proteins are altered qualitatively but not quantitatively by DDC. **A** Gross image of liver harvested from wild type mice fed with regular diet (RD) and 2 weeks of DDC diet. **B** Hepatic total protein level in 9-week-old mice wild type mice fed with RD and 2 weeks of DDC diet. **C** Total liver lysates from 9-week-old mice were analyzed by CBB staining. Protein band intensities were quantified by densitometry. **D** Liver sections were stained for p62/SQSTM1 and Ubiquitin (Ub). Arrows indicate hepatocytes without aggregated p62 or Ub. Scale bars: 10 μm. **E** Immunostained for p62 in primary hepatocytes isolated from 9-week-old wild type mice fed with RD and 2 weeks of DDC diet. **F** Quantitative PCR analysis for p62, K8, and K18 mRNA expression in 9-week-old mice wild type mice fed with RD and 2–4 weeks of DDC diet. The mRNA expression levels were normalized to actin. **G** Total liver lysates from 9-week-old mice were analyzed by immunoblotting for K8/18, p62 and GAPDH. Data are expressed as the mean ± SEM. n.s not significant, ^*^P ≥ 0.05, ^**^P ≥ 0.01, ^***^P ≥ 0.001 (*n* = 3). **H** Liver sections were co-stained for K8/K18 and p62. Arrows indicate hepatocytes without aggregated p62. Scale bars: 10 μm.

In order to determine the potential role of cell survival mechanisms in DDC mediated liver injury, we used an acute model of DDC intoxication. DDC diet was acutely fed to wild-type mice for 2 weeks to assess how hepatic protein would change. The gross examination revealed enlarged and dark brown livers, which are characteristic of DDC (Fig. 1A). In contrast, total hepatic protein levels did not differ significantly between control and DDC diet-fed mice (Fig. 1B). In addition, Coomassie Brilliant Blue staining (CBB) did not demonstrate significant differences in liver protein content (Fig. 1C). CBB patterns were quite different in the DDC-exposed liver, indicating a qualitative, but not quantitative, change in the hepatic proteome.

To analyze the qualitative change in the hepatic protein, immunofluorescence staining for p62/Sequestosome-1 was performed. p62/Sequestosome-1 is a major component of IHBs [5]. P62 binds Ubiquitin (Ub) and acts as an adapter linking ubiquitinylated species to other proteins. Hence p62 acts as a common denominator in a variety of cytoplasmic inclusions. The p62 and Ub proteins were co-localized and markedly elevated in immunofluorescence staining (Fig. 1D). A similar accumulation of p62 was observed in primary hepatocytes isolated from mice fed a DDC diet for 2 weeks (Fig. 1E). Interestingly, there was no notable change in mRNA expression for p62 (Fig. 1F). p62 and Ub aggregates can cross-link with the intermediate filament proteins keratins 8 and 18(K8/K18) to form MDBs [12]. The expression levels of K8 and K18, were elevated at both mRNA and protein levels (Fig. 1F, G). Increased levels of both normal and high-molecular-weight (HMW) size of K8 and K18 were observed in DDC-exposed liver (Fig. 1F, G).

We then examined whether p62-Ub containing IHBs contain the K8/K18 proteins, given that IHB formation generally results in MDB formation [12], and we found a significant elevation of K8/K18 expression in the DDC diet-treated condition (Fig. 1G). Despite immunoblotting showing an elevated level of HMW K8/K18, immunofluorescence staining of K8/K18 did not reveal protein aggregates (Fig. 1H, Supplementary Fig. 1). Additionally, the K8/K18 aggregates did not co-localize with the p62 aggregates, suggesting acute DDC causes p62 protein aggregates but not K8/K18 aggregates. Thus, acute DDC causes a qualitative change in the hepatic proteome and likely sets the stage for aberrant cellular stress signaling. As a result of acute DDC, p62-Ub inclusion bodies are formed without cytokeratins, suggesting they are IHBs instead of MDBs.

### DDC deregulates the hepatic protein chaperones

The liver accumulates p62-Ub IHBs after acute DDC exposure (Fig. 1). Hepatic IHBs could form selectively due to a failure of the molecular chaperone. During cellular stress, heat shock proteins (HSPs) serve as molecular chaperones that guide conformations necessary for protein synthesis, folding, translocation, and assembly [13, 14]. HSP70/90 keep newly formed misfolded proteins in a folding-competent state until the physiological situation improves during cellular stress. Furthermore, Hsp70 can refold misfolded proteins in ATP-dependent manner. A previous study identified Hsp25 and Hsp70 as MDB components [15]. When proteins are misfolded and then aggregate in various organelles, they can lead to proteotoxicity. Accordingly, we hypothesized that deregulated HSPs contribute to improper protein folding, resulting in protein accumulation and then proteotoxic liver injury in the acute DDC-diet model. Therefore, we examined the effect of DCC-diet on molecular chaperonin expression.

**Fig. 2:**
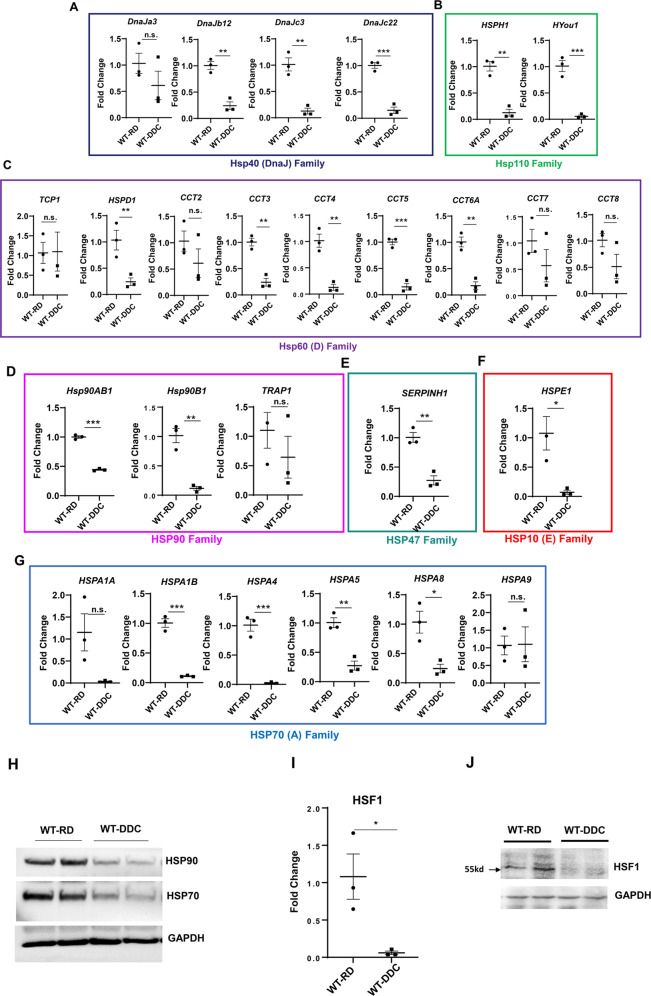
Liver protein chaperonin system is suppressed by DDC. Quantitative PCR analysis for **A** Hsp40 (DnaJ) Family, **B** Hsp110 Family, **C** Hsp60 Family, **D** HSP90 Family, **E** HSP47 Family, **F** HSP10 **E** Family, **G** HSP70(A) Family Hsp’s mRNA expression in 9-week-old mice wild type mice fed with RD and 2 weeks of DDC diet. The mRNA expression levels were normalized to actin. **H** Total liver lysates from 9-week-old mice were analyzed by immunoblotting for HSP90, HSP70, and GAPDH. **I**–**J** HSF1 protein and mRNA expression in 9-week-old mice wild-type mice fed with regular diet (RD) and 2 weeks of DDC diet. Data are expressed as the mean ± SEM. n.s not significant, *P ≥ 0.05, **P ≥ 0.01, ***P ≥ 0.001 (*n* = 3).

There are seven major families of HSPs: HSP110, HSP90, HSP70(A), HSP60(D), HSP40(DnaJ), HSP47, and HSP10(E) [13]. In the DDC-diet fed liver, we observed decreased expression of nearly all the 26 HSPs across all major heat shock families (Fig. 2A, G). Members of the HSPs family are found in the cytosol, mitochondria, and endoplasmic reticulum (ER) [16]. All HSPs were downregulated, regardless of cellular location (Supplementary Fig. 2). A downregulation of the HSPs’ mRNA was reflected appropriately in their protein levels (Fig. 2H).

Transcriptional activation of HSPs is orchestrated by heat shock factor 1 (HSF1), which translocate rapidly to nucleus and promotes HSP gene expression [16]. We hypothesized that HSF1 signaling is directly involved in suppressing HSPs in DDC-exposed livers. The DDC diet also suppressed the mRNA and protein levels of HSF1 (Fig. 2I, J, Supplementary Fig. 3) suggesting that the proteostasis signaling pathway is being deregulated in DDC diet-fed livers. Interestingly, refeeding regular diet to DDC fed mice restored HSF1 and HSP expression (Supplementary Fig. 4), suggesting that suppression of molecular chaperonins and HSF1 is temporary. Thus, acute DDC can suppress the protein molecular chaperone system, resulting in the formation of p62-Ub-IHBs. Additionally, the downregulation of these proteostasis factors may contribute to selective protein accumulation.

### Autophagy flux is impaired by an acute DDC diet

Inhibited clearance of protein aggregates by autophagy and ubiquitin-proteasome degradation pathways could also result in hepatic IHB formation [17, 18]. At basal levels, hepatocytes have active autophagy and can use it to counteract proteotoxicity. In addition, p62 protein, which is one of the selective autophagic substrates, accumulates in the livers of DDC-exposed mice (Fig. 1D, E). It is unclear whether impaired hepatic autophagy contributes to IHB formation in the DDC diet-exposed mice. Next, we examined the levels of autophagy-specific markers LC3-I and LC3-II in DDC, and regular diet fed mice.

**Fig. 3:**
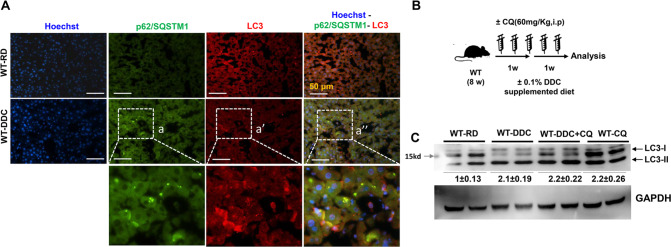
Autophagy flux is impaired by DDC. **A** Co-immunostained for p62 and LC3 in liver section prepared from wild type mice fed with regular diet (RD) and 2 weeks of DDC diet. The a, a’, and a” represents the enlarged images from the respective images from DDC diet fed wild type liver. Scale bars: 50 μm. **B** Schematics of CQ administration to Regular diet (RD) or 2 weeks of DDC diet fed wild type mice. **C** Total liver lysates were analyzed by immunoblotting for LC3, and GAPDH. Protein band intensity was normalized to GAPDH band intensity. Data are expressed as the mean±SEM.

DDC-fed mice showed an increase in LC3-positive autophagic puntae (Fig. 3A, Supplementary Fig. 5). Immunofluorescence staining revealed co-localization of LC3 with p62 autophagic substrate (Fig. 3A), suggesting that DDC diet alters liver autophagy. The increased intracellular autophagic puntae could result either from greater autophagy induction or from impaired autophagosome-lysosome fusion [19]. To check these possibilities, we performed autophagic-flux in DDC-fed mice. Autophagy flux is measured by turnover of LC3-II which is formed by conjugation of phosphatidylethanolamine (PE) to the LC3-I form [19]. LC3-II selectively localized to the autophagosomes membrane and is degraded in the lysosome with the fusion of autophagosomes with the lysosomes [19]. So, autophagy flux is often inferred based on LC3-II turnover, measured by immunoblot in both the presence and absence of lysosomal degradation. Chloroquine (CQ) is an established and popular inhibitor of lysosomal degradation. As an autophagy flux inhibitor, CQ was intraperitoneally administered to DDC diet-fed mice (Fig. 3B). CQ treatment (lane 7th and 8th) (Fig. 3C) significantly increased the protein level of both LC3-I and II compared to the regular diet control (lane 1st and 2nd) suggesting that the CQ is effective in blocking the autophagic degradation of LC3-II. DDC treatment itself increases LC3-II level(lane 3rd and 4th), but the DDC plus CQ treatment (lane 5th and 6th) does not increase LC3-II levels compared to CQ alone (lane 7th and 8th). Since there is no additive or supra-additive effect in LC3-II level in DDC plus CQ treatment, (when compared to CQ only group), it indicates that the DDC induced partial block in autophagy flux [19]. These results indicate that increased autophagosome punctae are likely due to DDC-mediated impairment of autophagosome-lysosome fusion. Overall, these data suggest that hepatic IHBs formation (protein accumulation) is not only due to downregulation of the HSF1 and HSPs signaling networks, but also to impaired autophagy clearance of IHBs. It is unclear whether these two cellular events are mechanistically linked.

### Impaired autophagic flux is not due to endoplasmic reticulum (ER) stress signaling

The DDC diet causes impaired hepatic autophagy by blocking the fusion of autophagosomes with the lysosomes and preventing the degradation of p62-Ub IHBs (Fig. 3). Autophagic flux impairment is observed during xenobiotic or superfluous nutrient stress such as during thapsigargin treatment and high fat-fed conditions [20–22]. Thapsigargin treatment induces ER stress by inhibiting sarcoendoplasmic reticulum calcium transport ATPase (SERCA) and blocks autophagosome-lysosome fusion [20]. Similarly, free fatty acid *(*FFA) from a high-fat diet induces ER stress, inhibits SERCA activity- increasing cytosolic calcium in hepatocytes that lead to the inhibition of autophagic function primarily by blocking the fusion step [22]. A DDC diet could also induce ER stress due to protein misfolding and aggregate formation [3].

**Fig. 4:**
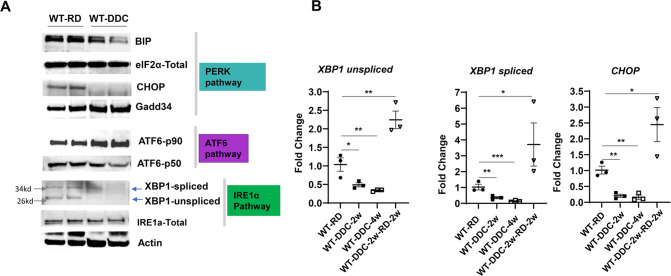
Inhibition of autophagic flux by DDC diet is not triggered by ER stress. **A** Total liver lysates were analyzed by immunoblotting for PERK Pathway (BIP, eIF2a, CHOP, GADD34, ACTIN), ATF6 pathway (ATF-p90, ATF-p50, ACTIN), and (IRE1a pathway (XBP1-spliced, XBP1-unspliced, ACTIN) proteins. **B** Quantitative PCR analysis for XBP1-uncut, XBP1 spliced, and CHOP mRNA expression from wild type mice fed with regular diet (RD), 2–4 weeks of DDC diet, and 2 week DDC followed by RD. The mRNA expression levels were normalized to actin. Data are expressed as the mean±SEM. ^*^P ≥ 0.05, ^**^P ≥ 0.01, ^***^P ≥ 0.001 (*n* = 3).

We then asked whether the DDC induces ER stress to impair hepatic autophagy. We examined the status of various ER stress markers in livers exposed to the DDC diet. When misfolded proteins accumulate, three constitutive client proteins (PKR-like endoplasmic reticulum kinase (PERK), inositol-requiring enzyme-1α (IRE1α), and activating transcription factor 6 (ATF6)) are activated, which act as upstream activators of coordinated signal transduction called unfolded protein response (UPR) [23]. Examination of various signaling proteins concerning the PERK pathway (eIF2α, CHOP, GADD34), ATF6 pathway (ATG6-p90, ATG6-p50, and IRE1α pathway (XBP1-spliced, XBP1-unspliced, IRE1α) did not show any remarkable upregulation of these proteins level in DDC exposed liver lysate. Instead, their expression levels were downregulated (Fig. 4A). Also, DDC exposure resulted in significant lower mRNA expression levels of XBP1 or CHOP (Fig. 4B). It is interesting to note that the suppression of these ER stress proteins can be reversed by refeeding regular diet after 2-weeks of DDC exposure (Fig. 4B). The DDC diet suppresses ER stress rather than activating it. These results suggests that ER-stress is unlikely to mechanistically explain the observed autophagic flux impairment in the DDC exposed liver.

### Impaired autophagic flux is correlated to the downregulation of Rab proteins

**Fig. 5:**
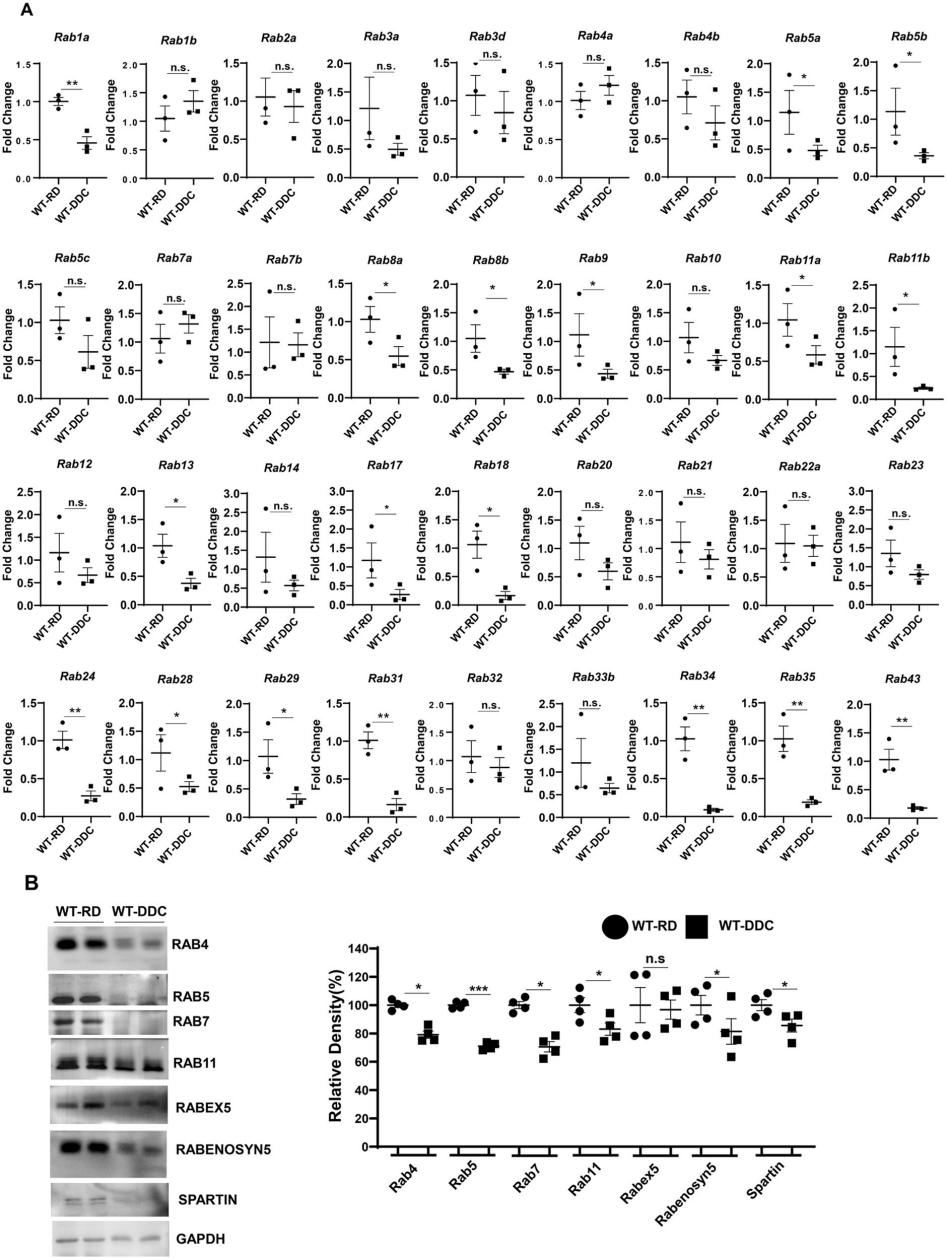
Inhibition of autophagic flux by DDC diet may be due to the downregulation of Rab proteins. **A** Quantitative PCR analysis for Rab family genes in mRNA expression in wild type mice fed with regular diet (RD), and 2 weeks of DDC diet. The mRNA expression levels were normalized to actin. Data are expressed as the mean±SEM. n.s not significant, ^*^P ≥ 0.05, ^**^P ≥ 0.01, ^***^P ≥ 0.001 (*n* = 3). **B** Total liver lysates were analyzed by immunoblotting for RAB4, RAB5, RAB7, RAB11, RABEX5, RABENOSYN5, SPARTIN and GAPDH proteins. Protein band intensity was normalized to GAPDH band intensity. Data are expressed as the mean±SEM. n.s not significant, ^*^P ≥ 0.05, ^***^P ≥ 0.001 (*n* = 2).

The autophagic degradation process involves vesicular formation, transport, tethering, and fusion [24]. The autophagic flux particularly involves the fusion between autophagosomes and lysosomes for the purpose of degrading enclosed contents, such as protein aggregates. Rab protein is a small GTPase of the Ras-like GTPase superfamily and shuttles between GTP-bound active and GDP-bound inactive states to facilitate vesicular trafficking [25]. Rab small GTPases have been found to participate in the autophagic process because autophagy involves vesicular trafficking [26]. It has been shown that Rab1, Rab5, Rab7, Rab9A, Rab11, Rab23, Rab32, and Rab33B participate in autophagosomes [24]. In particular, Rab7 is implicated in the fusion of autophagosomes with lysosomes [27–30]. Therefore, we examined whether impaired autophagic flux in DDC-exposed liver could be related to deregulated Rab GTPases. We compared the expression levels of Rab genes in the DDC diet-fed liver with those of the regular diet-fed liver. In rodents, 59 Rab genes are expressed at detectable levels, and we selected 36 of these Rab genes for quantitative PCR analysis. We focused on Rabs implicated in autophagy or expressed in livers. There was a general downregulation of Rab genes in livers exposed to the DDC diet (Fig. 5A). This observation was confirmed by analyzing the protein expression levels of a few Rabs and Rab-associated proteins, such as Rabex5, Rabenosyn5, and Spartin (Fig. 5B, C). These data suggest that suppression of Rab GTPase expression and function may impair autophagic flux and cause qualitative changes in the hepatic proteome of DDC exposed liver.

### DDC-mediated autophagy impairment activates p62-NRF2 and mTORC1 signaling

Due to DDC exposure, hepatic autophagy is impaired, resulting in accumulation of p62 (Fig. 1D, E, H). In addition to being an autophagy substrate, hepatic p62 can also serve as a signaling hub [31]. The p62 is a stress-inducible and multifunctional protein that contains several protein-protein interaction domains-a Phox1 and Bem1p (PB1) domain, a zinc finger (ZZ), two nuclear localization signals (NLSs), a TRAF6 binding (TB) domain, a nuclear export signal (NES), and LC3-interacting region (LIR), a Keap1-interacting region (KIR), and a ubiquitin-associated (UBA) domain [32, 33]. These p62’s protein domains can interact with various binding partners to activate downstream signaling pathways and may play a pathogenic role in DDC exposed liver. As a next step, we looked at possible signaling pathways, including the NRF2 pathway, the mammalian target of rapamycin complex 1 (mTORC1), and nuclear factor kappa B (NF-kB), that might have a pathological impact on DDC-exposed livers.

**Fig. 6:**
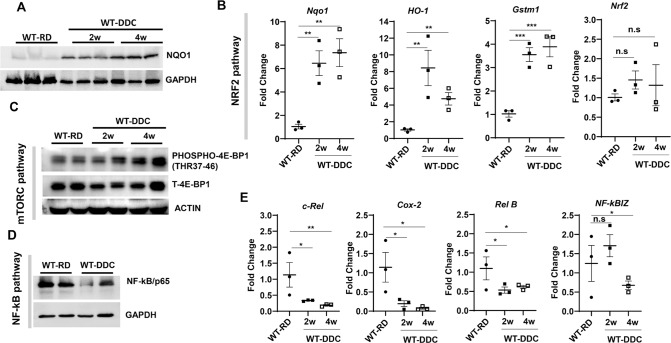
Autophagy impairment is linked to activation of the NRF2 and mTORC1 signaling pathways, not to the NF-kB signaling pathway. **A** Total liver lysates were analyzed by immunoblotting for p65/NF-kB and GAPDH proteins in wild type mice fed with regular diet (RD), and 2 weeks of DDC diet. **B** Quantitative PCR analysis for c-Rel, COX2, Rel-B and NF-kBIZ mRNA expression in wild type mice fed with RD, and 2–4 weeks of DDC diet. The mRNA expression levels were normalized to actin. Data are expressed as the mean±SEM. n.s not significant, ^*^P ≥ 0.05, ^**^P ≥ 0.01, *n* = 3). **C**, **D** Total liver lysates were analyzed by immunoblotting for phospho-4E-BP1, Total 4E-BP1, and ACTIN proteins and NQO1, and GAPDH in wild type mice fed with RD, and 2–4 weeks of DDC diet. **E** Quantitative PCR analysis for Nqo1, HO-1, Gstm1, and Nrf2 mRNA expression in wild type mice fed with RD, and 2–4 weeks of DDC diet. Data are expressed as the mean±SEM. n.s not significant, ^**^P ≥ 0.01, ^***^P ≥ 0.0001 (*n* = 3).

Accumulation of p62 can activate NRF2 by a non-canonical pathway [34, 35]. The p62 protein competitively binds to Kelch-like-ECH-associated protein 1 (KEAP1), an adaptor component for Cullin3-based E3 ubiquitin ligase complex that ubiquitinates NRF2 for proteasomal degradation. Moreover, NRF2 is an anti-oxidative stress-activated transcription bZIP factor, that can promote the transcriptional expression of K8/K18 expression [36]. On the other hand, DDC-mediated inhibition of ferrochelatase causes accumulation of PP-IV that can cause oxidative stress [37] and hence could activate NRF2. Next, we analyzed the expression of NRF2’s downstream target protein NQO1 in DDC-exposed livers. The immunoblot analysis of DDC exposed liver showed a marked elevation of NQO1 (Fig. 6A). As NRF2 is activated, it translocates to the nucleus where it binds to anti-oxidative response elements present in the promoter regions of antioxidative response genes such as NQO1, HO-1, and Gstm1. PCR analysis of the DDC-exposed liver also demonstrated that downstream target genes NQO1, HO-1, and Gstm1 were significantly upregulated (Fig. 6B). Notably, the NRF2 mRNA expression did not change, suggesting that NRF2 protein accumulation and its activation is via non-canonical p62 accumulation as observed in the DDC diet exposed liver.

p62 can promote mTORC1 activation by directly interacting with Raptor, a key component of mTORC1 [33]. The region between the ZZ and TB domains (amino acids 167–230) of p62 is required for the interaction between p62 and Raptor [33]. Furthermore, over-expression of p62 enhanced mTORC1 activation [33]. Immunoblot analysis of mTORC1 downstream target protein showed elevated levels of phosphorylated-4E-BP (Fig. 6C) suggesting that p62 accumulation can activate the mTORC1 pathway in the DDC diet. Activation of the mTORC1 pathway could further suppress autophagy, resulting in a vicious cycle.

p62 regulates NF-kB by interacting with αPKC through its PB1 domain, RIP through its ZZ domain, and TRAF6 through its TB domain [38]. As such, the p62 protein interacts with RIP and bridges RIP to αPKCs, resulting in the activation of NF-kB by the TNFα signaling pathway [32]. Interestingly, the expression level of TNFα has been reported to be elevated in DDC-exposed conditions [6]. As a result, the NF-kB pathway may be activated in livers exposed to DDC. Therefore, we examine whether p62 accumulation causes activation of NF-kB in DDC-exposed livers. Immunoblot analysis showed a lower level of p65/NF-kB protein in DDC diet-fed mice (Fig. 6D). NF-kB downstream target genes, such as c-Rel, Cox2, Rel B, or NF-kBIZ, were also significantly downregulated (Fig. 6E), suggesting that acute exposure to DDC suppresses rather than activates the NF-kB pathway.

This study clearly demonstrates that autophagy impairment in the DDC diet causes accumulation of p62, which activates the NRF2 and mTORC1 signaling pathways. DDC treatment does not activate the NF-kB signaling pathway, but rather suppresses it.

### Impaired autophagy by DDC is associated with suppression of hepatic FXR signaling and deregulated bile acid (BA) metabolism

We previously found that autophagy-deficiency in hepatocytes led to an intracellular cholestasis with increased serum and hepatic BA levels [39]. Upon autophagy-deficiency, p62 accumulates and activates the non-canonical NRF2 pathway, which then transcriptionally represses the expression of the FXR nuclear receptor, a master regulator of hepatic BA metabolism [39]. Several studies have established that DDC-exposed livers display BA metabolic disturbances and intrahepatic cholestasis. Due to DDC’s impairment of hepatic autophagy and activation of NRF2, we examined whether impaired NRF2 activity in DDC could also be related to FXR downregulation and other BA metabolism abnormalities.

**Fig. 7:**
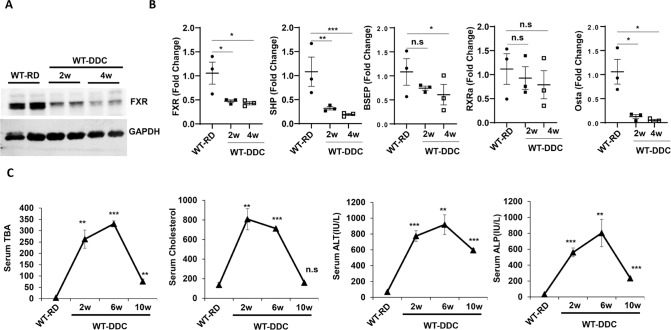
DDC repress hepatic FXR signaling and impairs bile acid handling. **A** Total liver lysates were analyzed by immunoblotting for FXR and GAPDH proteins in wild type mice fed with regular diet (RD), and 2–4 weeks of DDC diet. **B** Quantitative PCR analysis for FXR, SHP, BSEP, RXRα, and OSTα mRNA expression in wild type mice fed with RD, and 2–4 weeks of DDC diet. The mRNA expression levels were normalized to actin. Data are expressed as the mean±SEM. n.s not significant, ^*^P ≥ 0.05, ^**^P ≥ 0.01, ^***^P ≥ 0.0001, (*n* = 3). **C** Serum Total Bile Acid (TBA), Cholesterol. ALT, and ALP levels were quantified for 9-week-old mice fed with RD, and 2–10 weeks of DDC diet. Data are expressed as the mean±SEM. n.s not significant, ^**^P ≥ 0.01, ^***^P ≥ 0.0001 (*n* = 3).

The FXR protein level in DDC exposed liver was significantly lower than in wild type control liver (Fig. 7A). The mRNA levels of FXR and its downstream targets, SHP, BSEP, and Ostα, were also significantly downregulated (Fig. 7B). The mRNA expression level of RXRα, a binding partner of FXR, did not change significantly in the DDC exposed liver (Fig. 7B). A dramatic increase in serum levels of Total Bile Acid (TBA), cholesterol, triglycerides, Total Bilirubin (TB), Direct Bilirubin(DB), and liver injury markers-ALT and ALP was observed in the mice exposed to DDC (Fig. 7C, Supplementary Fig. 6A, B). Interestingly the cholestatic liver injury parameters were partially recovered with prolonged DDC exposure suggesting the development of a compensatory resistance mechanism in the liver (Fig. 7C, Supplementary Fig. 6A, B). Expression analysis of various BA transporters showed elevation of Apical (MDR1A, MDR1B) and systemic (Ostβ, Mrp3, Mrp4) BA transporters. The expression levels of basolateral or enterohepatic (NTCP, OATP2, OATP1, and OATP4) BA transporters were suppressed (Supplementary Fig. 7) indicating the compensatory adaptation in responses to cholestasis. These results suggest that DDC leads to downregulation of FXR nuclear receptors and cholestatic liver injury. An activation of the p62-NRF2 signaling pathway in the DDC diet may suppress FXR and cause cholestatic liver damage.

### Impaired hepatic autophagy exacerbates DDC induced cholestatic liver injury

**Fig. 8:**
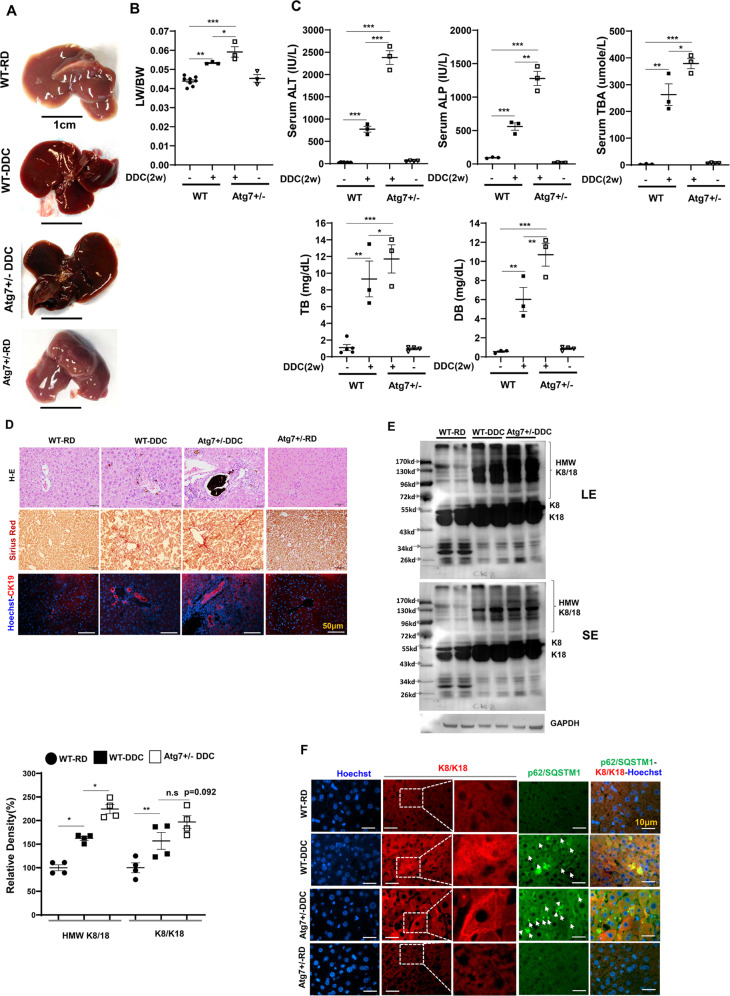
Impaired hepatic autophagy exacerbates DDC mediated cholestatic liver injury. **A** Representative gross morphology of liver of regular diet (RD) or DDC diet fed wild type mice and 2-week DDC diet fed Atg7 + /- mice. **B** LW/BW ratio showing hepatomegaly in the 2 weeks DDC diet fed wild type or Atg7 + /- mice. **C** Serum ALT, ALP, Total Bile Acid (TBA), Total bilirubin (TB), and direct bilirubin (DB) levels were quantified in the 2 weeks DDC diet fed wild type or Atg7 + /- mice. Data are expressed as the mean±SEM. n.s not significant, ^**^P ≥ 0.01, ^***^P ≥ 0.0001 (*n* = 3). **D** Liver sections were subjected to H&E staining (original magnification, ×200), Sirius Red stain (original magnification, ×200), and immunostained for CK19 to detect Ductular cells (original magnification, ×200). Scale bars: 50 μm (CK19). **E** Total liver lysates were analyzed by immunoblotting for K8/18, and GAPDH proteins in the 2 weeks DDC diet fed wild type or Atg7 + /- mice. Protein band intensity was normalized to GAPDH band intensity, LE long exposure, SE short exposure. Data are expressed as the mean±SEM. n.s not significant, ^*^P ≥ 0.05, ^***^P ≥ 0.001 (*n* = 2). **F** Liver sections were co-stained for K8/K18 and p62. Scale bars: 10 μm.

Based on the above observation, we investigated whether impairment of autophagy due to DDC exposure could exacerbate cholestatic liver injury. We fed DDC diet to Atg7 + /- mice for two weeks. The Atg7 gene is essential for the formation of autophagosomes [2]. We choose hepatocyte specific Atg7 + /- in place of Atg7-/- mice for DDC treatment because Atg7-/- develop cholestatic liver injury on its own [39, 40]. Furthermore heterozygous deletion of Atg7 results in accumulation of LC3-I/II and p62(HMW) in stressed condition like DDC diet feeding (Supplementary Fig. 8A), suggesting partial impairment in autophagy function.Atg7 + /- exposed to DDC showed enlarged and dark brown livers like their wild-type counterparts (Fig. 8A). Hepatomegaly induced by DDC was further exacerbated in Atg7 + /- livers (Fig. 8B). More importantly, the DDC exacerbated the cholestatic liver injury as examined by serum ALT, ALP, TBA, TB, DB in Atg7 + /- mice (Fig. 8C, Supplementary Fig. 8B). Atg7 + /- mice also showed increased ductular reaction when compared to DDC fed wild type mice (Fig. 8D, Supplementary Fig. 8C). Further, K8/18 proteins were also further accumulated in Atg7 + /- mice fed DDC (Fig. 8E). In Atg7 + /- mice, immunofluorescence staining showed a distinct, bright filamentous staining pattern running along the cell periphery (Fig. 8F). This K8/K18 immunostaining pattern is distinctly different when compared to diffused staining pattern of DDC exposed wild type liver (Fig. 8F). Therefore, genetic impairment of autophagy function exacerbates cholestatic liver injury induced by DDC.

## Discussion

MDBs are commonly seen in hepatocellular carcinoma, Wilson’s disease, non-alcoholic steatohepatitis, and other chronic liver disorders. The main components of MDBs are K8/18, p62, Ub, and chaperones [12]. While IHBs contain p62 and ubiquitin (although not constantly), they lack K8/K18 [12]. Incorporating “abnormal” keratins and HSPs into aggregated p62 causes p62-containing IHBs to become classical MDBs [12]. MDBs can be experimentally induced in the livers of mice chronically fed DDC [5]. The cellular status of these established MDB proteins is less clear in the acute model of DDC intoxication. We show that an acute DDC exposed liver has an increased Ub and p62 positive protein aggregates, as seen in autophagy-deficient livers (Fig. 1D, F, H) [41]. A CBB staining shows that high molecular weight (HMW) cytosolic proteins are prominent in DDC-exposed liver despite no significant differences in overall hepatic protein levels((Fig. 1B, C). Autophagic clearance impairment and improper chaperonin handling likely contribute to HMW protein accumulation in the liver of mice exposed to DDC diets.

**Fig. 9:**
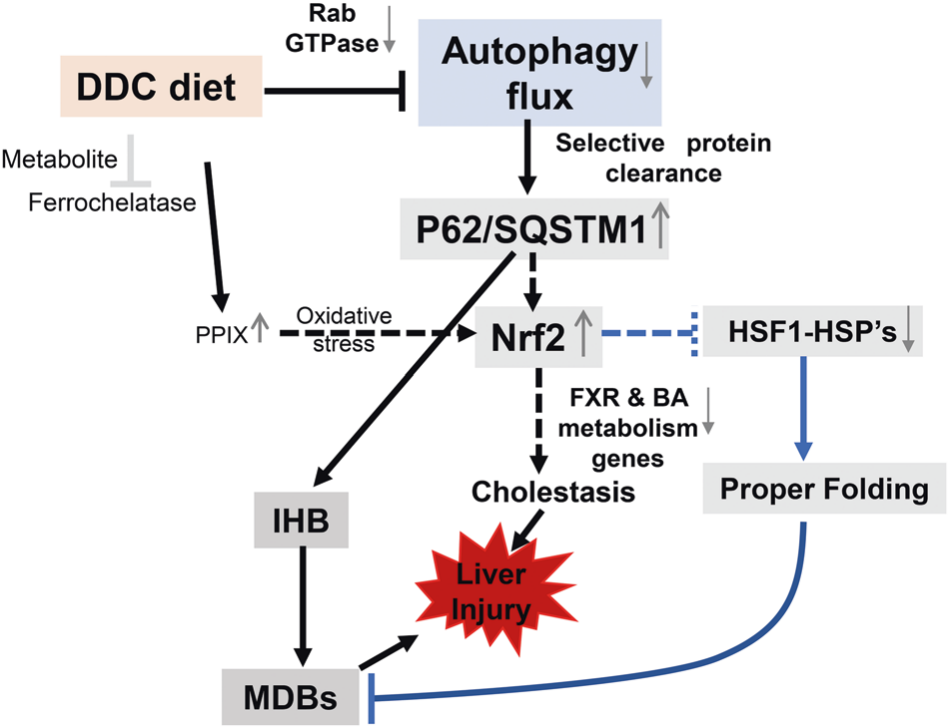
Schematic model showing role of autophagy in DDC diet. Acute DDC impairs the autophagic flux due to Rab suppression. Impaired autophagy leads to formation of p62 containing intrahyaline bodies (IHB). Impaired autophagy can also relate to downregulation of hepatic protein chaperonin system, further aiding in IHB formation. Further activation of P62 related NRF2 can suppress the FXR nuclear receptor and cause cholestatic liver injury. Unknown mechanism is represented by a broken line.

There is a significant decrease in various HSPs involved in regulating protein conformation in the livers of DDC-intoxicated mice (Fig. 2A–J). The MDBs were not formed in acute DDC intoxicated livers, despite elevated K8/K18 protein levels and the presence of p62-Ub-containing IHBs. A downregulation of HSPs may explain the lack of MDB formation at the onset of DDC intoxication. It is unclear why so many HSPs have been downregulated in the livers of mice exposed to DDC diets. In situ promoter analysis with MatInspector and JASPER revealed a putative anti-oxidative response element (ARE) binding site for NRF2 in HSF1, the master regulator of HSP expression (Katherine B et al. unpublished data). There is a possibility that activating NRF2 might suppress HSF1 and inhibit the expression of HSPs (Fig. 9).

Rab proteins belong to the Ras superfamily of small G proteins. They regulate vesicular traffic by switching between GTP-bound and GDP-bound conformations. GDP-bound Rab is inactive, whereas GTP-bound Rab is active. GTP/GTP exchange factors (GEPs), GDP dissociation inhibitors (GDIs), and GTPase-activating proteins (GAPs) regulate the conversion between states. There are distinct subcellular locations where Rab proteins can be found. The Rab7 is present in autophagosomes [24, 27] and promotes microtubule plus-end-directed transport and fusion of autophagosomes with lysosomes through a novel FYVE and coiled-coil domain-containing protein FYCO1 [25]. Rab7 is also involved in maturation of late autophagic vacuoles, recruitment of dynein/dynactin motors, Rabring7 (Rab7-interacting ring finger protein), and hVPS34/p150 complexes [26]. Thus, decreased Rab protein expression may contribute to impaired autophagosome and lysosomal fusion in DDC.

There is no clear explanation for why the DDC diet suppresses Rab proteins so strongly. There is a possibility that DDC suppresses Rab upstream regulators. In addition, liver-derived DDC-related metabolites may inhibit Rab expression. DDC N-alkylates the heme of certain hepatic cytochrome-p450 enzymes to produce N-methyl protoporphyrin (NMPP). NMPP is a potent inhibitor of ferrochelatase which is involved in the conversion of porphyrinogen IX (PP-IX) into heme, thus resulting in the accumulation of PP-IX and upstream porphyrin intermediates [37]. The accumulation of NMPP or PP-IX could be responsible for the downregulation of Rab proteins. Future studies will examine the common motifs in the Rab genes’ promoters and the transcription factors that control their expression.

DDC diet suppresses ER stress signaling despite the accumulation of intrahepatic proteins. DDC or liver-derived DDC-related metabolites perturb porphyrin metabolism at multiple steps, leading to the accumulation of N-methyl protoporphyrin-IX (NMPP) and porphyrinogen IX (PP-IX) [37]. ER stress signaling may be suppressed by the accumulation of these porphyrin intermediates. As observed in this study, DDC diets also significantly downregulate most protein chaperones. The ER stress signaling pathway is largely modulated by protein chaperones. Activation of the ER stress signaling pathway would become futile if HSF1 and HSPs chaperones are severely deregulated. It is also possible that protein accumulation would be more towards the cytosol than within ER lumens, which are generally responsible for activating ER stress signaling. Since ER stress signaling dampens protein accumulation stress, without ER stress signaling, protein stress could intensify and hence exacerbate the DDC-diet induced liver pathologies.

The hepatotoxin DDC may interfere with the autophagy mechanism in cells. According to our results, acute DDC exposure impairs autophagosome-lysosome fusion, resulting in p62 accumulation and IHB formation. As yet, it is unknown how exactly impaired autophagy flux results in cholestatic liver injury in mice fed DDC diets. It is important to note, however, that this study supports previous findings that a defect in autophagy caused accumulation of p62, activation of NRF2 and repression of FXR to result in cholestatic liver injury [39]. The autophagic pathway maintains the functional integrity of the FXR, and a deficit of autophagy suppresses the expression of the FXR through the activation of NRF2. As a result of p62 accumulation in the autophagy-deficient liver, NRF2 is activated and FXR is repressed, leading to cholestatic liver injury [39]. In the DDC model of hepatotoxin-induced liver injury, we confirm the pathological significance of the autophagy-p62-NRF2-FXR pathway. In another alternative, DDC can cause oxidative stress, activating NRF2 via conventional pathways by oxidizing cysteine residues in KEAP1. PP-IX-mediated oxidative stress may also inhibit NRF2 proteasomal degradation by DDC feeding [37].

Moreover, the role of autophagy in hepatotoxin-induced liver injury is unclear. Our data strongly suggest that impaired autophagy function can increase the susceptibility to hepatotoxin-induced cholestatic liver injury. Therefore, activating autophagy may be an effective therapeutic strategy for cholestatic liver injury induced by hepatotoxins.

## Materials & methods

### Animal experiments

All animals used in these experiments were treated with humane care, in accordance with the guidelines from the National Institutes of Health’s Guide for the Care and Use of Laboratory animals, and with approval from Institutional Animal Care and Use Committee (IACUC) of Tulane University. Wild type male mice(C57BL/6 J) were randomly assigned to the regular diet fed group and the DDC-diet fed group. Atg7 + /- mice ((C57BL/6 J background) were generated by crossing Alb-Cre mice and the Atg7 F/F control mice as described previously [9, 42].

### DDC diet

Adult mice of the same age (2 month, approximately 25 gram) was randomly assigned to either the regular diet group or the DDC diet group. Those in the regular diet group were fed a regular chow diet of commercially available mouse food pellets (free of xenobiotics) Mice in the DDC diet were fed a diet containing 0.01% of 3,5-diethoxycarbonyl-1,4-dihydrocollidine (DDC) for 2–10 weeks. The mice were then humanely sacrificed, and their livers were collected and frozen to be used for experimental preparations.

### Immunofluorescence microscopy

Paraffin sections were prepared from the liver samples using a microtome. After deparaffinization and rehydration, these sections were treated with a citrate buffer (pH 6.0) for antigen retrieval. The slides were then permeabilized and blocked with 5% donkey or goat serum in PBS with 0.1% Triton X and glycine for one hour. The slides were incubated at 4 °C in a solution of primary antibodies diluted in PBS (Supplementary antibody list Table 1). The slides were then washed in PBS with 0.1% Triton X and then incubated with a solution of PBS containing fluorophore-conjugated secondary antibodies for at least one hour. Nuclei were stained with Hoechst 33342 (1 μg/ml). Images were obtained using a Nikon Eclipse TE 200 epi-immunofluorescence microscope and the companion NIS-Elements AR3.2 software.

### Serum parameters analysis

Blood samples were collected, and serum analyzed for ALT, TBA, TB, TC, and TG using commercially available kits from Pointe Scientific, following the manufacturer’s protocol.

### Immunoblotting

Total liver protein lysate was prepared from the liver samples using RIPA lysis buffer containing protease inhibitor cocktail. The liver lysate was centrifugation after homogenizing the samples using an electric homogenizer. Sample total protein lysates’ concentrations were measured using a BCA assay (using commercially available kit following standard protocol) and then separated by sodium dodecyl sulfate polyacrylamide gel electrophoresis (SDS-PAGE). After SDS-PAGE, the proteins were transferred to PVDF membrane. The membranes were blocked in TBS with 0.1% Tween 20 (TBS-T) and 5% non-fat dry milk powder for at least one hour. The membranes were then incubated with primary antibodies (Supplementary antibody list Table 1) overnight at 4 °C. The membranes were washed with TBS-T and incubated with a horseradish peroxidase-conjugated secondary antibody for 1 h. Blots were visualized using the immunobilion chemiluminescence system (Millipore, MA) kit and BioRad chemiluminescence machine scanner. The densitometry analysis of immunoblot images was performed using quantity One Software (Bio-Rad). Densitometry values were normalized to the loading control (GAPDH or ACTIN) and then converted to unites relative to the untreated control.

### Quantitative PCR

A GeneJET RNA Purification Kit (Thermo Fisher Scientific) was used to extract the total RNA from homogenized liver samples, according to the manufacturer’s protocol. 1 μg total RNA was then used to synthesize cDNA using a M-MLV Reverse Transcriptase Enzyme System (Life Technologies, Thermo Fisher Scientific) and OligoT primers. qPCR was performed using SYBR Green Master Mixes on a Quanta studio 3 PCR machine (Life Technologies–Applied Biosystems, Thermo Fisher Scientific), using gene-specific primers included in the Supplementary primer list Table 2. Gene expression was calculated using the 2^–ΔΔCt^ method and normalized to the housekeeping gene Actin.

### Hematoxylin and Eosin (H&E) and Sirius Red staining

Slides with mouse liver tissue, sectioned in paraffin and fixed in formalin, were stained with H&E and Sirius Red according to standard protocol. Images were obtained using Olympus light microscope and analyzed using the companion NIS-Elements AR3.2 software.

### Statistical analysis

Data is presented in figures as the mean with error bars representing±SEM. SigmaStat 3.5 software was used to perform statistical analysis. Statistical analysis was performed using P values from at least 3 samples, calculated using a 2-tailed Student’s *t* test for paired group comparisons or 1-way ANOVA with appropriate post hoc analysis for multigroup data comparisons. The statistical analysis methods used were chosen appropriately for variance and distribution fitting. *P* values of less than 0.05 were considered statistically significant, denoted by “*”, while *p* values of less than 0.01 were denoted by “**”, and *p* values of less than 0.001 were denoted by “***”.

## Supplementary information

SUPPLEMENTARY FIGURES Legends

Supplementary Table 1-2

Supplementary Figure 1

Supplementary Figure 2

Supplementary Figure 3

Supplementary Figure 4

Supplementary Figure 5

Supplementary Figure 6

Supplementary Figure 6

Supplementary Figure 7

Supplementary Figure 8

## Data Availability

All experimental data generated and/or analyzed during the study are available from the Corresponding author on reasonable request.
