# Supplementary material for: Impaired hepatic autophagy exacerbates hepatotoxin induced liver injury
Source: Cell Death Discov. 2023 Feb 21;9:71. doi: 10.1038/s41420-023-01368-3 (PMC9944334; doi:10.1038/s41420-023-01368-3)
Supplement: Supplementary file 1 — SUPPLEMENTARY FIGURES Legends [file 41420_2023_1368_MOESM1_ESM.docx]

**SUPPLEMENTARY FIGURES**

**Supplementary Figure 1**. **Hepatic IHB formation in DDC diet exposed liver.** Liver sections were stained for K8 and K18. Scale bars: 10 μm

**Supplementary Figure 2**. **HSPs are downregulated regardless of subcellular localization.** (A) Quantitative PCR analysis for mRNA expression of HSP’s localized to endoplasmic reticulum (ER), nuclear membrane, vesicles, cytoplasm, nucleoplasm, ER that belongs to Hsp40 (DnaJ) Family, Hsp110 Family, HSP90 Family, or HSP47 Family. The cDNA was prepared from mRNA isolated from 9-week-old mice wild type mice fed with regular diet (RD) and 2 weeks of DDC diet. The mRNA expression levels were normalized to actin. Data are expressed as the mean ± SEM. n.s: not significant, *P≥0.05, **P≥0.01, ***P≥0.001 (n=3)

**Supplementary Figure 3**. **HSF1 and HSP’s remain suppressed in longer DDC fed liver.** Quantitative PCR analysis for HSF1, HSP90B1, and HSPA4 mRNA expression from 9-week-old mice wild type mice fed with regular diet (RD) or 2-6 weeks of DDC diet. The mRNA expression levels were normalized to actin. Data are expressed as the mean ± SEM. *P≥0.05, **P≥0.01, (n=3).

**Supplementary Figure 4**. **Hepatic suppression of HSF1 and HSP is reversible.** (A) Schematics of DDC diet recovery experimental model. (B) Quantitative PCR analysis for HSF1, HSP90B1, CCT8, HSPA4 and HSPA5 mRNA expression from 8-week-old mice wild type mice fed with either 4 weeks of DDC or 2 weeks DDC diet followed by 2 weeks Regular Diet (RD) The mRNA expression levels were normalized to actin. Data are expressed as the mean ± SEM. n.s: not significant, *P≥0.05, **P≥0.01, (n=3).

**Supplementary Figure 5**. **Autophagosomes are elevated in DDC diet exposed liver.** Autophagosomes detection by LC3 Immunostaining in liver section prepared from wild type mice fed with regular diet (RD) and 2 weeks of DDC diet. Scale bars: 10 μm.

**Supplementary Figure 6**. **DDC cause hepatomegaly and cholestatic liver injury.** (A) LW/BW ratio showing hepatomegaly in the DDC diet fed wild type mice for different time period. (B) Serum total bilirubin, Direct bilirubin and triglyceride levels were quantified for 9-week-old mice fed with regular diet (RD), and 2-10 weeks of DDC diet. Data are expressed as the mean ± SEM. n.s: not significant, **P≥0.01, ***P≥0.0001 (n=3).

**Supplementary Figure 7**. **Adaptation alteration in cholestasis associated bile transporters in DDC diet.** Quantitative PCR analysis for Apical Bile acid transporters (MRP2, MDR1A, MDR1B), Systemic BA transporters (OSTb, MRP3, and MRP4), and Basolateral/Enterohepatic BA transporters (NTCP, OATP2, OATP1, and OATP4) mRNA expression in wild type mice fed with regular diet (RD), and 2-4 weeks of DDC diet. The mRNA expression levels were normalized to actin. Data are expressed as the mean ± SEM. n.s: not significant, *P≥0.05, **P≥0.01, ***P≥0.0001, (n=3).

**Supplementary Figure 8**. **Cholestasis related parameters analysis in DDC diet exposed mice.** (A) Total liver lysates were analyzed by immunoblotting for p62, LC3, and GAPDH. (B) Serum total cholesterol (TC), and triglyceride levels were quantified in the 2 weeks DDC diet fed wild type or Atg7+/- mice. Data are expressed as the mean ± SEM. n.s: not significant, **P≥0.01, ***P≥0.0001 (n=3). (C) Liver sections were subjected to H&E staining (original magnification, 100X and ×200).
