# Supplementary material for: Impaired hepatic autophagy exacerbates hepatotoxin induced liver injury
Source: Cell Death Discov. 2023 Feb 21;9:71. doi: 10.1038/s41420-023-01368-3 (PMC9944334; doi:10.1038/s41420-023-01368-3)
Supplement: Supplementary file 2 — Supplementary Table 1-2 [file 41420_2023_1368_MOESM2_ESM.pdf]

Supplementary Table 1: RT-qPCR primers

Supplementary Table 1. Primers used in PCR assays

Gene Name	Forward Primer	Reverse Primer
BSEP	5'-CCTCTCACCAGGCTCTCTACC-3'	5'-CGCCACTGTGGAAAGTCAGGG-3'
CCT2	5'-TCACCACAAGGACCACTTTAC-3'	5'-CAGACTCCCACCTAGTTTCTTG-3'
CCT3	5'-GACCTGCTTGGGACCTAAAT-3'	5'-GGATGCTGGACTTGAATCTCT-3'
CCT4	5'-TCCTACTGTGTTCGTGCTTTC-3'	5'-GGCGGTTTCTTAGCTCTGTTA-3'
CCT5	5'-GCTGGGCTCCAAAGTGATTA-3'	5'-CAACATCTCTCCGCTCCATATC-3'
CCT6A	5'-GTCTACCCTTGTTCCGTAAGTG-3'	5'-CTGTAGCCTGAGTTGGCATAG-3'
CCT7	5'-CCGAGGCAAAGCAACAATATC-3'	5'-GGACTTGGCTATGTCCACTAAA-3'
CCT8	5'-CTGTGTACTCTTGTCCGTTTGA-3'	5'-TGAGGTTCTCCTCTCCCTTAC-3'
CHOP	5'-TCTGATTGACCGAATGGTGA-3'	5'-TCTGGGAAAGGTGGGTAGTG-3'
K18	5'-GGACATCGAGATCACCACCT-3'	5'-TGAAGCCAGGGCTAGTGAGT-3'
K8	5'-CAAGTCTGCCGAAATCAGGGAC-3'	5'-TCCAAGTTGATGTTCTGGTTTT-3'
COX2	5'-TGCAGAATTGAAAGCCCTCT-3'	5'-CCCCAAAGATAGCATCTGGA-3'
c-Rel	5'-AGTGACTCACCCCACCTCAC-3'	5'-AGGCCCTTCTAGGAATGGAA-3'
DnaJa3	5'-CATCCACTCGGACCTCTTTATTT -3'	5'-GCAGGGATCGTCACATTGAT-3'
DnaJb12	5'-GTCTCCAGGCTTATGGTTAAGG-3'	5'-TGTCTGGGATCTCTGCTGTA-3'
DnaJc22	5'-CTTGTCAGCACCATCCTCAA-3'	5'-CTCTACCCTTCTCATCCCTGTA-3'
DnaJc3	5'-AGCAAGGAGCTAACCGTATTT-3'	5'-CTGAACACCTCCACAGGATAAG-3'
FXR	5'-GGCCTCTGGGTACCACTACA-3'	5'-TGTACACGGCGTTCTTGGTA-3'
Gstm1	5'-ACTTGATTGATGGGGCTCAC-3'	5'-TCTCCAAAATGTCCACACGA-3'
HO-1	5'-GTGATGGAGCGTCCACAGC-3'	5'-TTGGTGGCCTCCTTCAAGG-3'
HSF1	5'-GTTCCAGCATCCTTGTTTCTTG-3'	5'-GACACTGTCCTGGCGTATTT-3'
HSP47= SERPINH1	5'-CATCTTCCTGGTGCGAGATAA-3'	5'-CCACTCTTGGACTCTACAACTC-3'
Hsp90AB1 = HspC3	5'-GCTATCCCATCACCCTCTATTT-3'	5'-GCTTCTCCTCATCCTCCTTATC-3'
Hsp90b1 = HspC4	5'-GCCCTCAAGGACAAGATAGAAA-3'	5'-TGTTGCCAGACCATCCATAC-3'
HSPA1A	5'-TGGTTGCACTGTAGGACTTG-3'	5'-CGAGTTCAGGATGGTTGTGT-3'
HSPA4	5'-TCTGAGCAGTCCATCCTTAGTA-3'	5'-GAACTCTCATCCTGTCCCATTC-3'
HSPA5	5'-GAGACTGCTGAGGCGTATTT-3'	5'-TGACATTCAGTCCAGCAATAGT-3'
HSPA8	5'-ACTCCTCTTTCCCTTGGTATTG-3'	5'-GTCAGAGTAGGTGGTGAAAGTC-3'
HSPA9	5'-ACTCCTGTGTGGCTGTTATG-3'	5'-GTCGTTCTCCATCTGCTGTAA-3'
HSPD1	5'-AGGTTGTGAGAACTGCCTTAC-3'	5'-TCCAGGGTCCTTCTCTTCTT-3'
HSPE1	5'-ACTGTAACCAAAGGTGGCATTA-3'	5'-GGCTCAATCTCTCCACTCTTTC-3'

Hsph1	5'-TCTTCAGTGTGGAGCAGATAAC-3'	5'-GAAGAAGGATGGGACTGAGATG-3'
HYou1	5'-CCCAGAATCTGACCACAGTAAA-3'	5'-GCCACTCTCATCCAGGTTAAA-3'
MDR1A	5'-AAAGGCTCTACGACCCCCTA-3'	5'-CCTGACTCACCACACCAATG-3'
MDR1B	5'-TTGGTGGCACAACAACTCAT-3'	5'-GGCTTTCGCATAGTCAGGAG-3'
MRP2	5'-GCACTGTAGGCTCTGGGAAG-3'	5'-TGCTGAGGGACGTAGGCTAT-3'
Mrp3	5'-GGACTTCCAGTGCTCAGAGG-3'	5'-AGCTGTGGCCTCGTCTAAAA-3'
Mrp4	5'-TGTTTGATGCACACCAGGAT-3'	5'-GACAAACATGGCACAGATGG-3'
Nf-KBIZ	5'-GTGGAGGCAAAGGATCGTAA-3'	5'-TCACGAAAGACAGGCAACTG-3'
Nqo1	5'-AGCGTTCGGTATTACGATCC-3'	5'-AGTACAATCAGGGCTCTTCTCG-3'
NTCP	5'-CACCATGGAGTTCAGCAAGA-3'	5'-CCAGAAGGAAAGCACTGAGG-3'
OATP1	5'-ATCCAGTGTGTGGGGACAAT-3'	5'-GCAGCTGCAATTTTGAAACA-3'
OATP2	5'-TTGCTGACTGCAACACAAAG-3'	5'-TGGTTCCAGTTCCAACAGAC-3'
OATP4	5'-TGGGATTCCATTCACTGGTT-3'	5'-TGCTCCACAGCTGGTTACAG-3'
Osta	5'-GTCTCAAGTGATGAACTGCCA-3'	5'-TTGAGTGCTGAGTCCAGGTC-3'
Ostb	5'-GTATTTTCGTGCAGAAGATGCG-3'	5'-TTTCTGTTTGCCAGGATGCTC-3'
p62/SQSTM1	5'-GCTCAGGAGGAGACGATGAC-3'	5'-AGAAACCCATGGACAGCATC-3'
Rab 13	5'GAGATCGGGAACCAACAGTAAG -3'	5'-GGGTGAATAGGAGGCAAGAAA-3'
Rab10	5'-AACCTCCTAACCTGGATTTGAC-3'	5'-CCCACTCCTTCTTGCTCTTT -3'
Rab11b	5'-CCCAGCTCTCGAACTCTTATTC-3'	5'-CAGAAGCTGAGTGGTAGGTTTC-3'
Rab12	5'-AAGGAGACGTTCGATGACTTG-3'	5'-CTGTCTCACAGTCCAGCTTATT-3'
Rab14	5'-CACACACGGAAATAAGACACAAC-3'	5'-CAGGAGCTCTTTCCAGCATTA-3'
Rab17	5'-CTGTAATCACTGCTTGCCAAAG-3'	5'-TCTGGGTATCAGGTAAGGTAGG-3'
Rab18	5'-GCATCCCAGAACTCACCTAAA-3'	5'-GCTTGATTCTGGAGCCTCTATC-3'
Rab1b	5'-CATGGCATCATTGTGGTGTATG-3'	5'-TGTTGCCTACCAGGAGTTTATT-3'
Rab20	5'-GGCCGCTATCATCCTTACATAC -3'	5'-CAGTCATTGTTGGCTGTTTCTG-3'
Rab21	5'-GGAGCCAAGCATTACCATACT-3'	5'-CTGGGCTGTCTCTATCATTCTTT-3'
Rab22a	5'-CAGCTTCCGCCATCTCTAAA-3'	5-CGTGGGTTCTGACACATACA-3'
Rab23	5'-CCGACAGGTAGATGAAAGGAATG-3'	5'-GCTTACAGTGGCTATGGAGAAG-3'
Rab28	5'-GCTCTGCTCCTTCATCCTTTAG-3'	5'-ATGGACTCTCCATTCGGATTTC-3'
Rab29	5'-CACATCCATGACACGACTCTAC -3'	5'-GTCCAGATCCTGTTTCCATCTT-3'
Rab2a	5'-GCAGGAGTCCTTTCGTTCTATC-3'	5'-GTTGAACGTGTCTCTCCTTGT-3'
Rab31 (Rab22b)	5'-CTCAAGACCATCAGTGCCTATC-3'	5'-TCAGCTCAATCGTTCTCTGTTT-3'
Rab34	5'-CTCCTGCTCAGTATTCCCTAATG-3'	5'-CTGCCACACGGAAGAAGAA-3'
Rab35	5'-CCACATCGGGCTCAGTATTT-3'	5'-ACAGGTATGAGGGTGCAAAG-3'
Rab3a	5'-CACCATCACCACAGCCTATT-3'	5'-GCATTGTCCCACGAGTAAGT-3'
Rab3d	5'-GTGTAGAAACGGAAGTGGAGAA -3'	5'-CAAGAGTCCTCATGTGGAGAAG -3'

Rab43	5'-CACACCATGAGGGCTGTATT-3'	5'-GTTCTGTTCCACTCCAGGTTAG-3'
Rab4b	5'-CCTACCTGAAGAACTCCCAAAG-3'	5'-GAACAGGCCTCCAGGTAATAAA-3'
Rab5b	5'-CAATGACAGGGCAGCTAGAA-3'	5'-CAGACACCCTCAAACACCTAATA-3'
Rab5c	5'-CAATGACCCGACTGGAATCTAC-3'	5'-CCGGCCTAGGATCAAAGTTATG-3'
Rab7b	5'-TGTCTTCACACTGCACAGATAG-3'	5'-GTGGAAGGCATGAGGTATGAA-3'
Rel B	5'-CTTCCAGCTTCCTCATCCTG-3'	5'-CCTCTTCGGACTCAGCATTC-3'
RXRa	5'-AGCCATTGTCCTGTTCAACC-3'	5'-CCTAGGTGGCTTGATGTGGT-3'
SHP/Nr0b2	5'-CTGGTTGAGCGCCTGAGAC-3'	5'-CTGCCTGGATGCCCTTTATC-3'
TCP1	5'-GCCTTGGGTGTCTCACATTA-3'	5'-CAGTGCAACACTAAGCAGAAAG-3'
TRAP1 = HspC5=HSP75	5'-CCAGTGATGCCTTGGAGAAA-3'	5'-GCCAGTGTCCTGAATGGTAATA-3'
XBP spliced	5'-GAGTCCGCAGCAGGTG-3'	5'-GTGTCAGAGTCCATGGGA-3'
XBP uncut	5'-GAATGGACACGCTGGATCCT-3'	5'-GCCACCAGCCTTACTCCACTC-3'

Supplementary Table 2: List of antibodies-

Supplementary Table 2. Antibodies used in immunoassays and IFC

**Antibody/Species**	**Source/Catalog Number/Dilution**
Actin/Mouse	Cell signaling Technology/#3700/1:4000
AMPK/Rabbit	Cell signaling Technology/#2532/1:1000
ATF6/Rabbit	Santa Cruz/sc-22799/1:500
BIP/Rabbit	Cell signaling Technology/#3183/1:1000
CHOPP/Rabbit	Cell signaling Technology/#2895/1:1000
K18/TROMA-I/Rat	Developmental Studies Hybridoma Bank/ AB_531826/1:500
K19/Rat	Developmental Studies Hybridoma Bank/1DB-001-0000868971/1:200
K8/TROMA-I/Rat	Developmental Studies Hybridoma Bank/ AB_531826/1:500
elF2α/Rabbit	Cell signaling Technology/#9722/1:1000
FXR/Mouse	R&D Systems/PP-A9033A-00/1:1000
Gadd34/Goat	Santa Cruz/sc-8832/1:500
GAPDH/Mouse	Novus Bilogicals/NB300-21/1:3000
HSF1/Rabbit	Cell signaling Technology/#4356/1:1000
HSP70/Rabbit	Cell signaling Technology/#4872/1:1000
HSP90/Rabbit	Cell signaling Technology/#4874/1:1000
IRE1a/Rabbit	Santa Cruz/sc-20790/1:500
LC3/Rabbit	MBL/PM036/1:1000
NF-κB/p65/Rabbit	Cell signaling Technology/#8242/1:1000
Nqo1/Rabbit	Abcam/ab34173/1:3000
P62/SQSTM1/Mouse	Abnova/H00008878-M01/1:1000
Phospho-4E-BP1/Rabbit	Cell Signaling Technology/#9459/1:1000
Rab11/Mouse	BD Pharmigen/610656/1:1000
Rab5/Mouse	BD Transduction/610724/1:1000
Rab7/Mouse	Sigma/R8779/1:1000
Rabenosyn 5/Goat	Abcam/ab21196/1:1000
Rabex-5/Mouse	BD Transduction/612558/1:1000
Spartin/Goat	Santa Cruz/sc-49521/1:500
Total-4E-BP1/Rabbit	Cell Signaling Technology/#9452/1:1000
XBP/Rabbit	Santa Cruz/sc-7160/1:500
Alexa-488-labeled anti-Rabbit 2nd Ab/Goat	InVitrogen/A-11034/1:500
Cy3-labeled anti-Rat 2nd Ab/Donkey	Jackson ImmunoResearch Laboratories Inc/712-165-150/1:500

HRP-labeled anti-Goat 2nd Ab /Donkey	Jackson ImmunoResearch Laboratories Inc/705-035-147/1:5000
HRP-labeled anti-Mouse 2nd Ab/Goat	Jackson ImmunoResearch Laboratories Inc/115-035-062/1:5000
HRP-labeled anti-Rabbit 2nd Ab/Goat	Jackson ImmunoResearch Laboratories Inc/111-035-045/1:5000
