# Supplementary figures and images for: Impaired hepatic autophagy exacerbates hepatotoxin induced liver injury
Source: Cell Death Discov. 2023 Feb 21;9:71. doi: 10.1038/s41420-023-01368-3 (PMC9944334; doi:10.1038/s41420-023-01368-3)

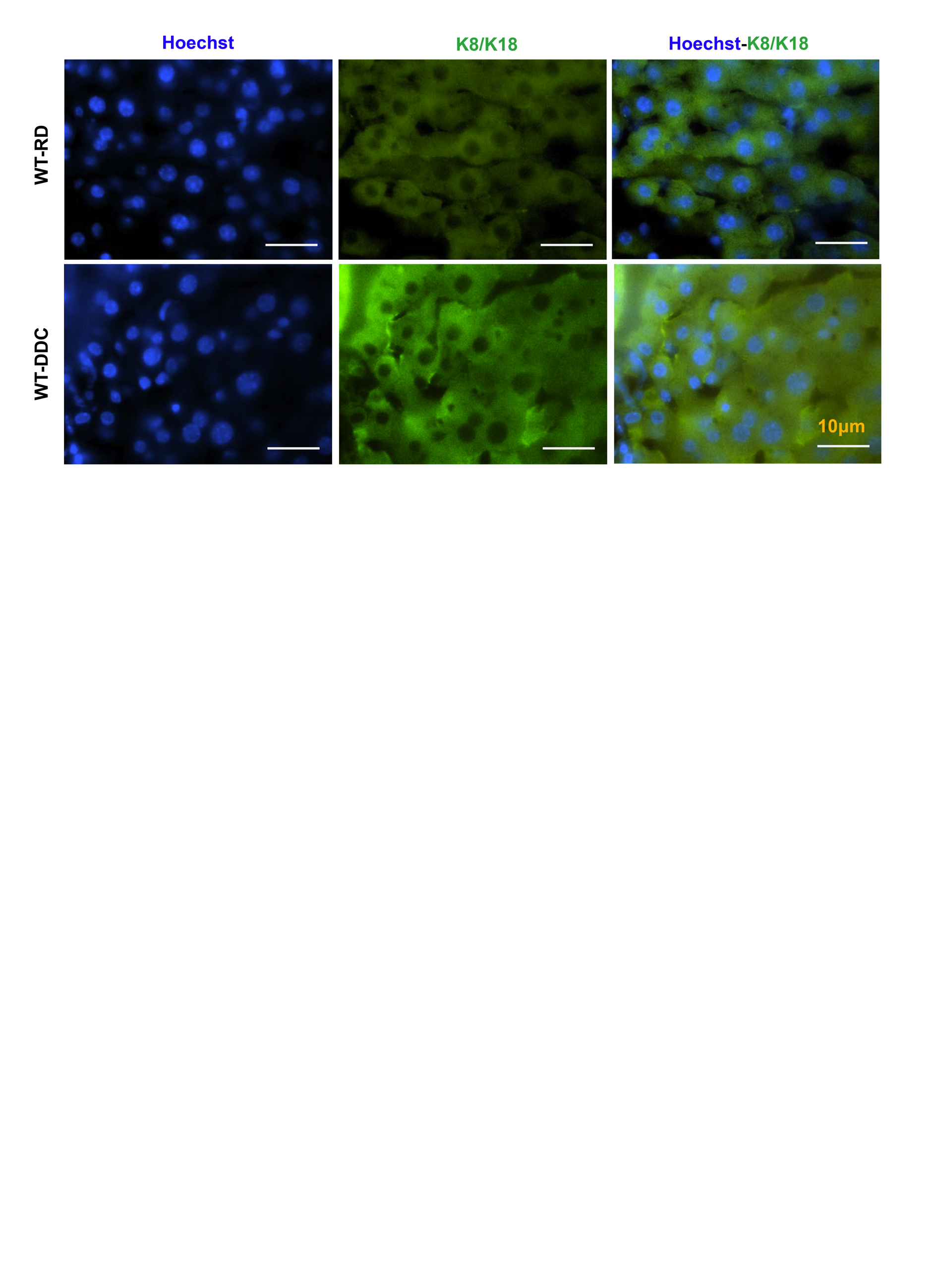

Supplement: Supplementary file 3 — Supplementary Figure 1 [file 41420_2023_1368_MOESM3_ESM.tif]

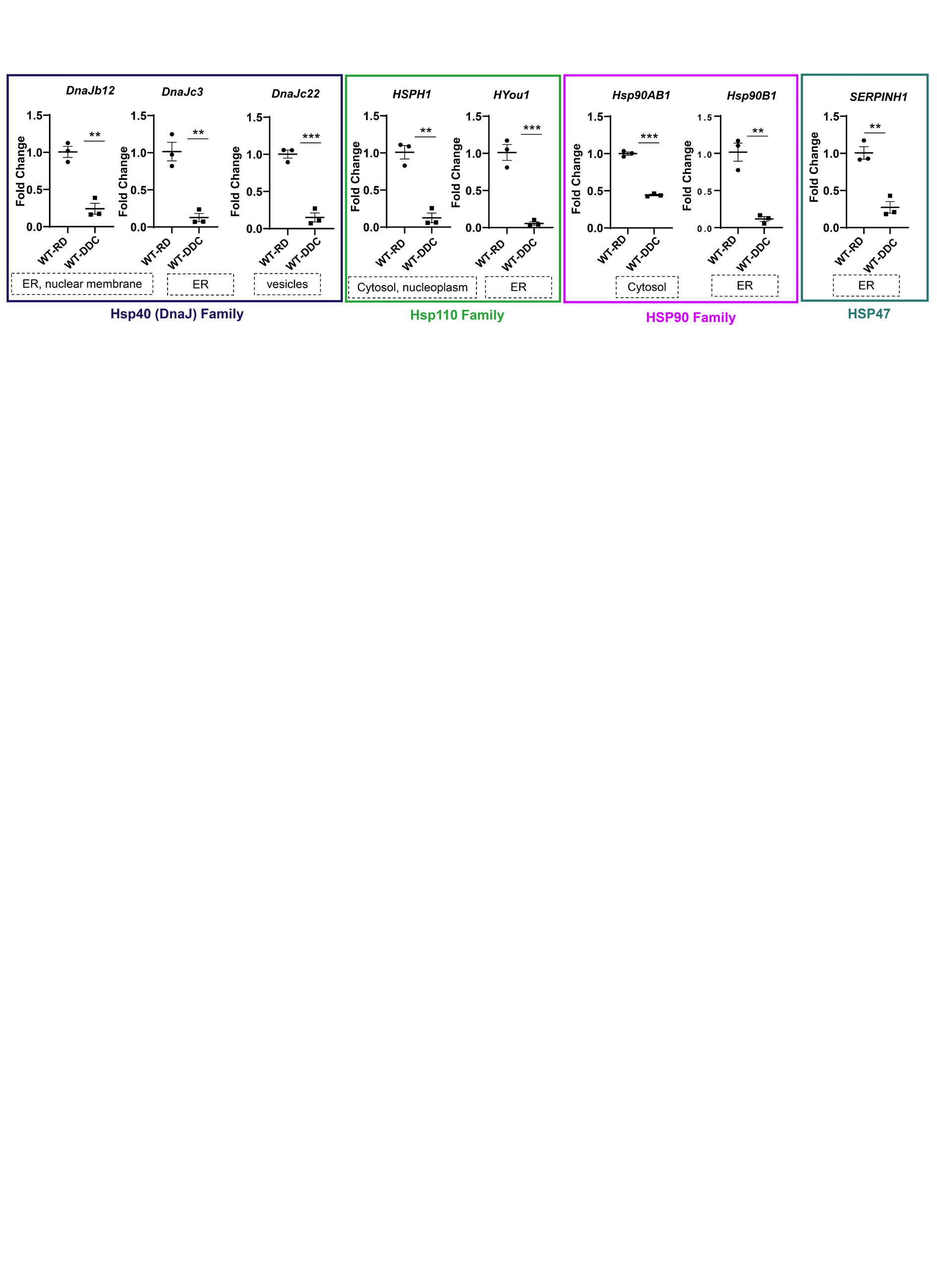

Supplement: Supplementary file 4 — Supplementary Figure 2 [file 41420_2023_1368_MOESM4_ESM.tif]

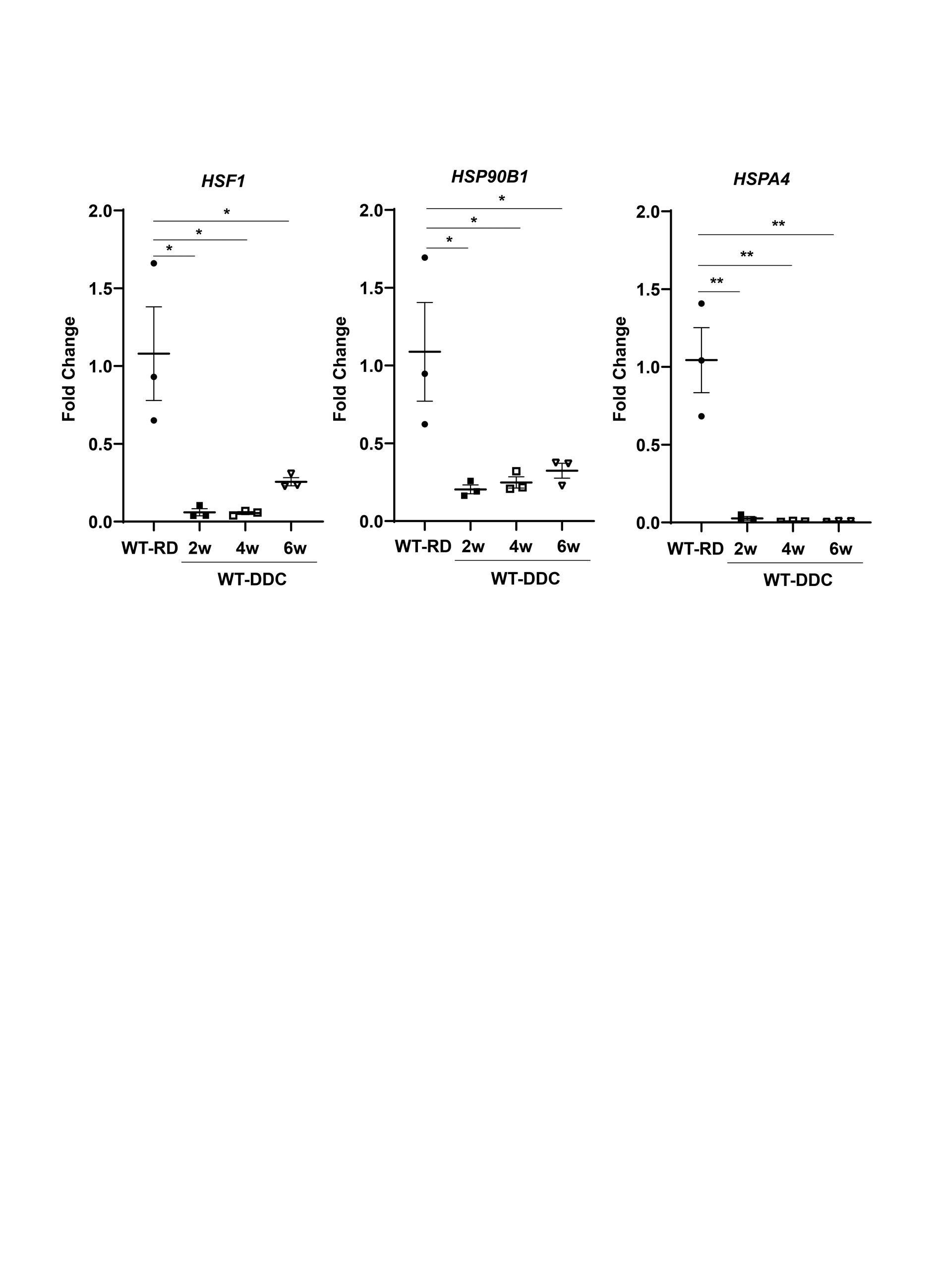

Supplement: Supplementary file 5 — Supplementary Figure 3 [file 41420_2023_1368_MOESM5_ESM.tif]

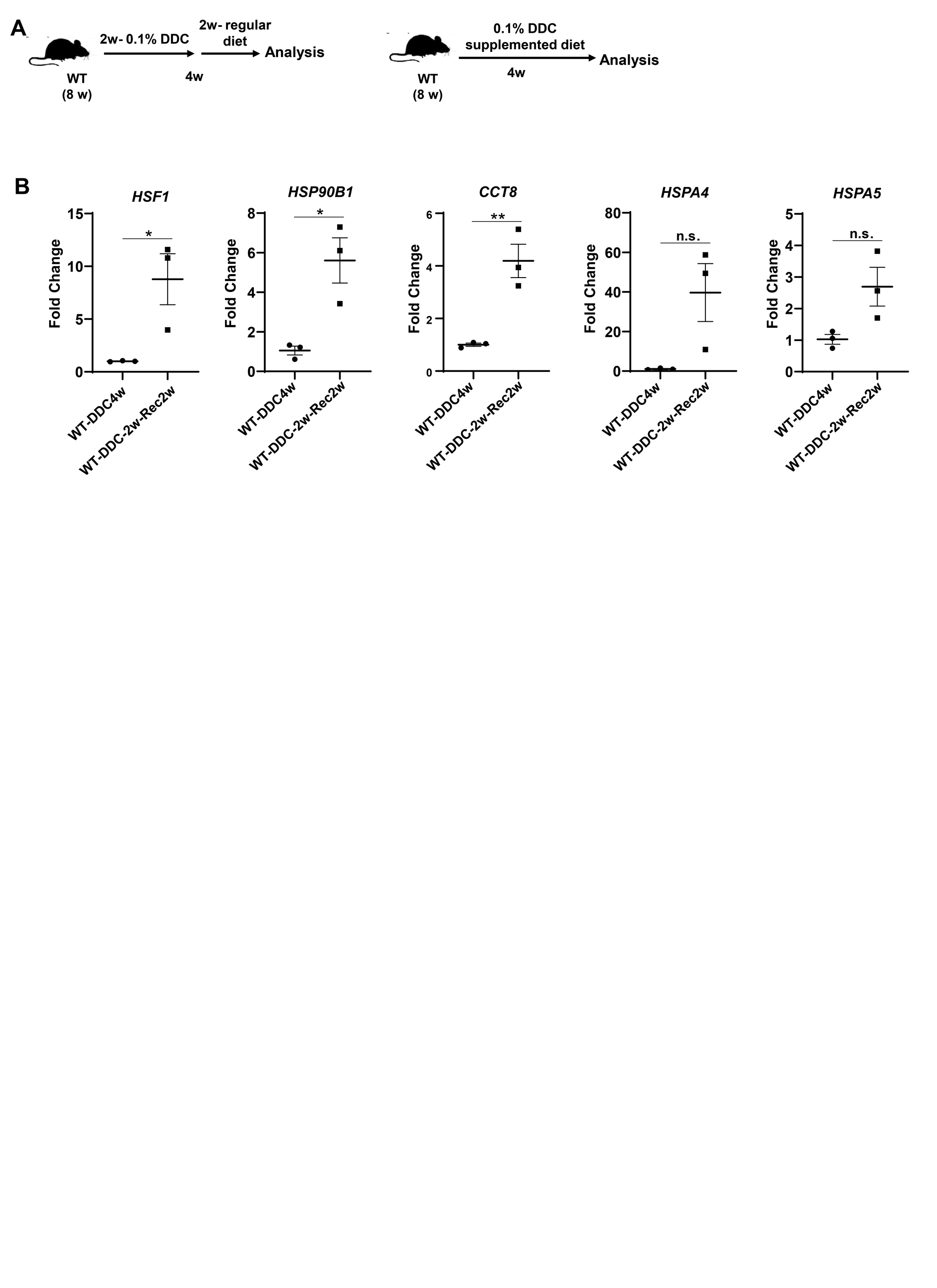

Supplement: Supplementary file 6 — Supplementary Figure 4 [file 41420_2023_1368_MOESM6_ESM.tif]

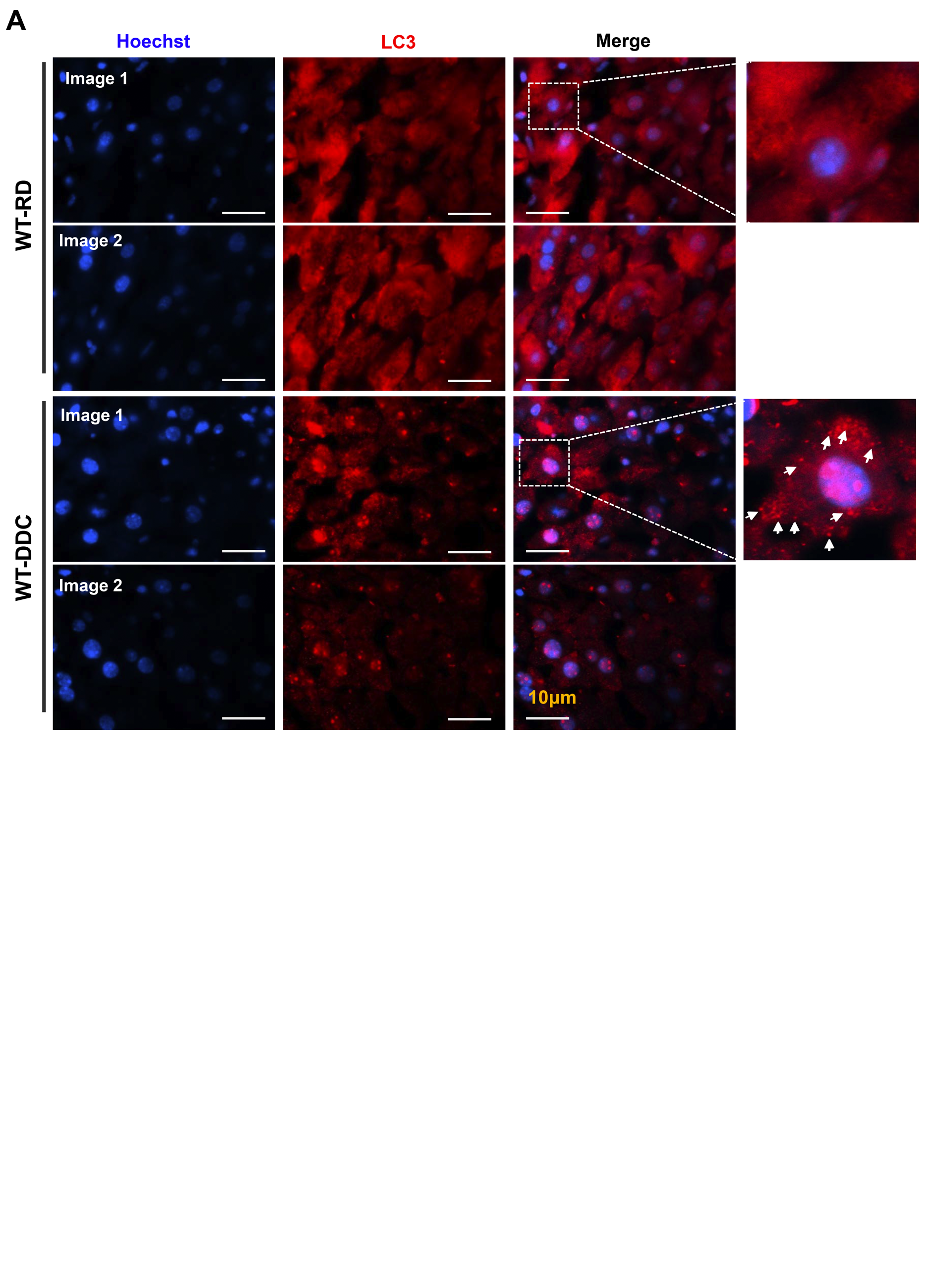

Supplement: Supplementary file 7 — Supplementary Figure 5 [file 41420_2023_1368_MOESM7_ESM.tif]

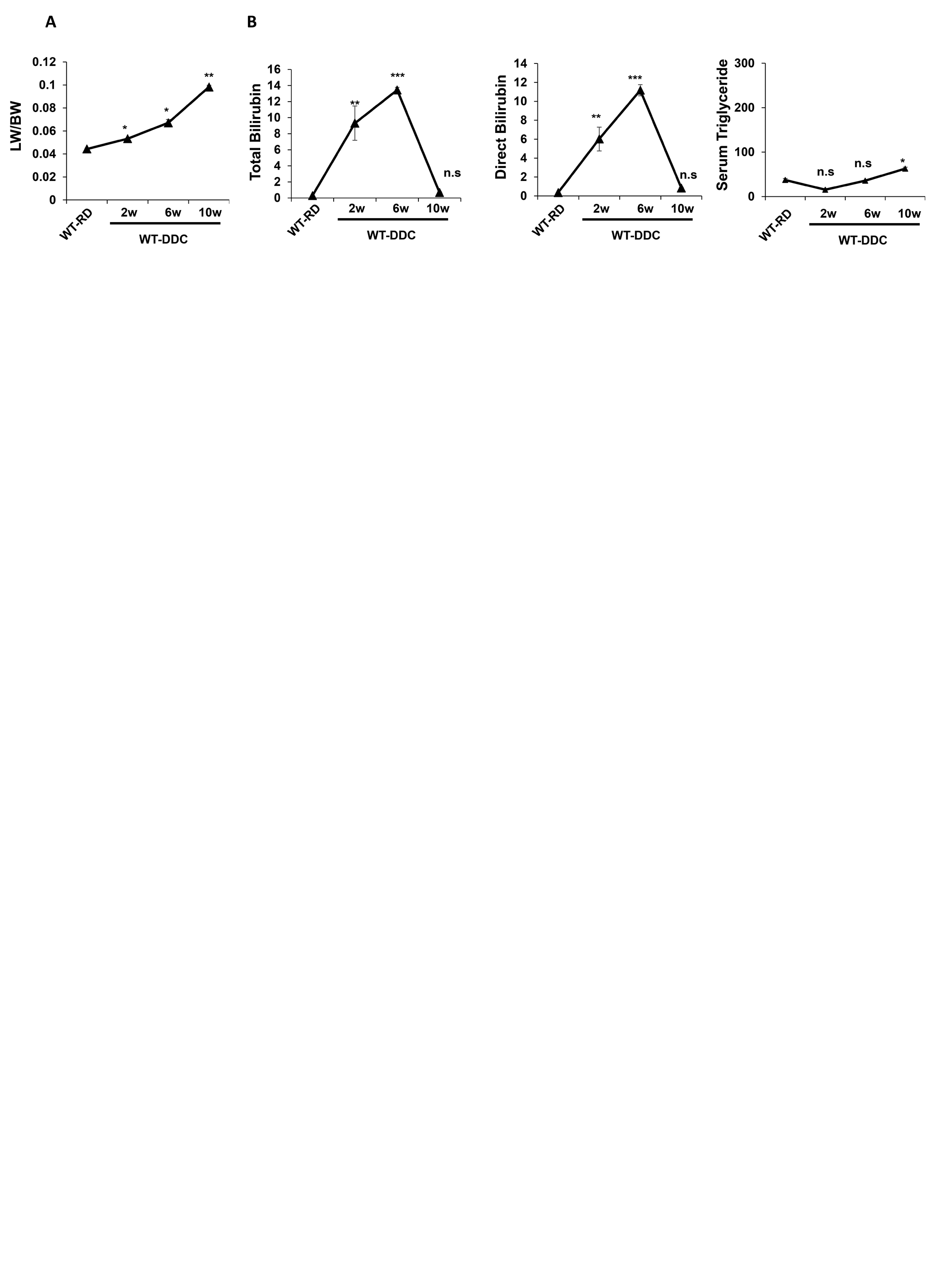

Supplement: Supplementary file 8 — Supplementary Figure 6 [file 41420_2023_1368_MOESM8_ESM.tif]

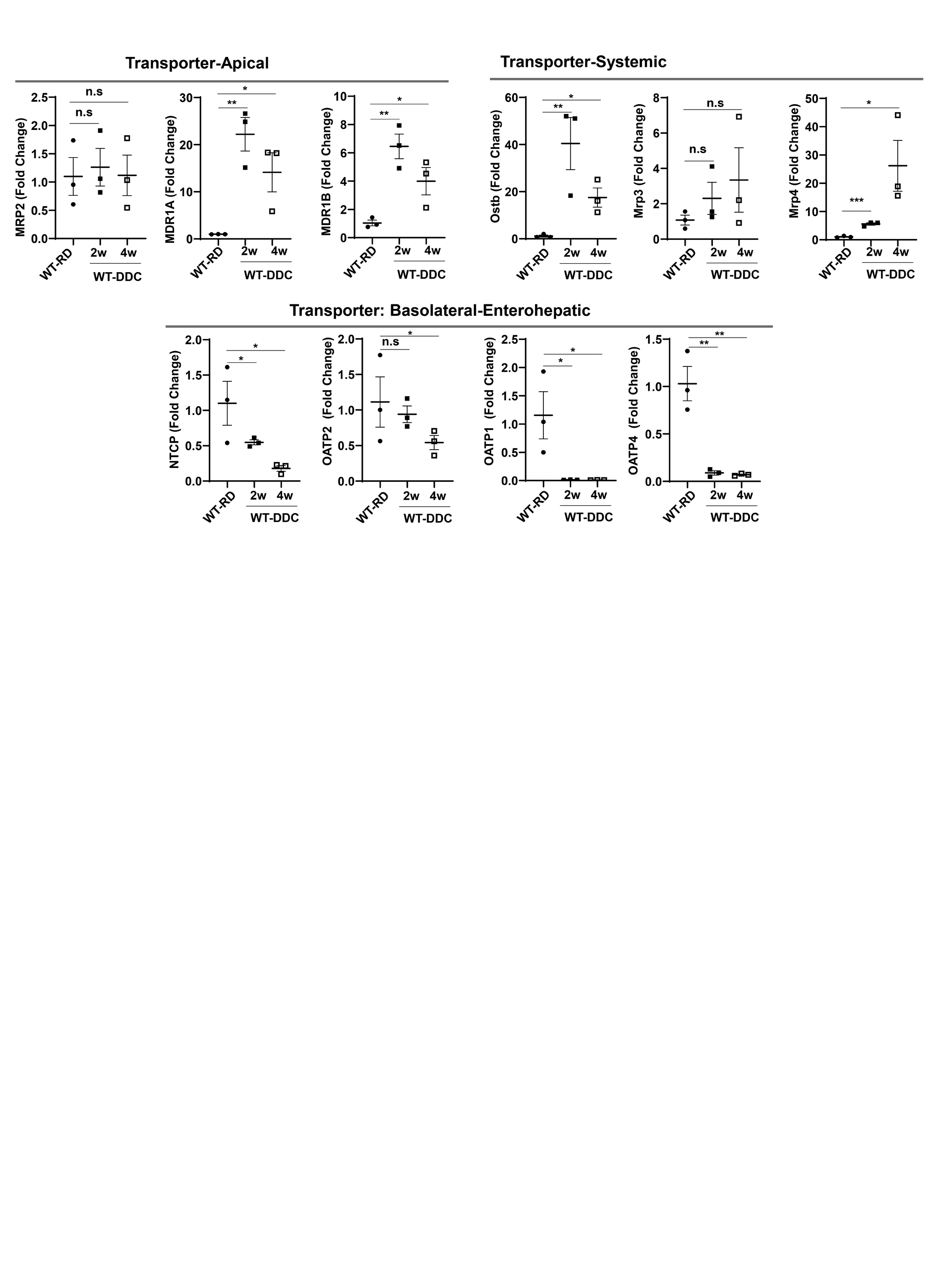

Supplement: Supplementary file 10 — Supplementary Figure 7 [file 41420_2023_1368_MOESM10_ESM.tif]

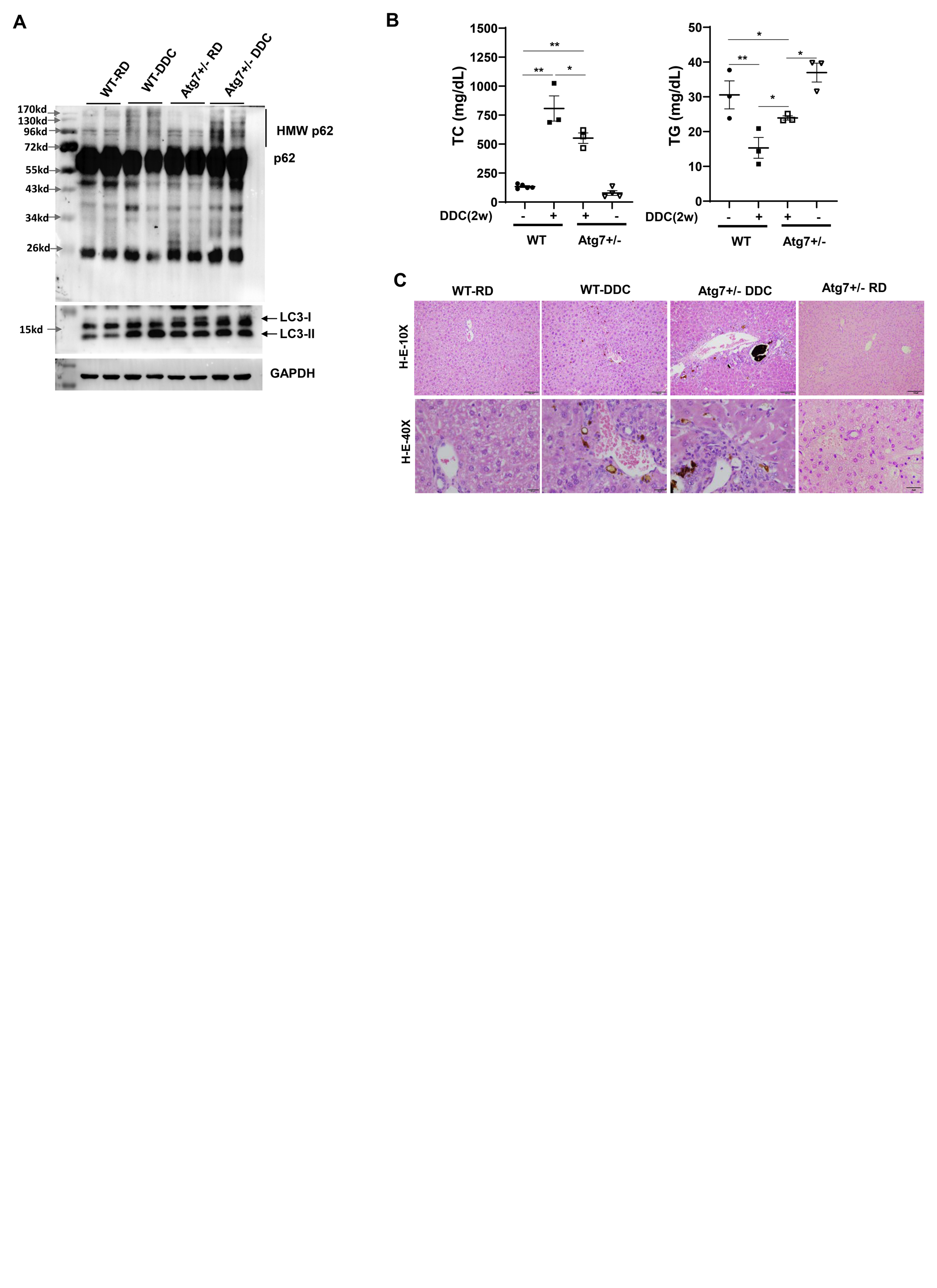

Supplement: Supplementary file 11 — Supplementary Figure 8 [file 41420_2023_1368_MOESM11_ESM.tif]
